# Integration of Untargeted Metabolomics, Network Pharmacology, Single-Cell RNA Sequencing, and Molecular Dynamics Simulation Reveals GOT1, CYP1A2, and CA2 as Potential Targets of Huang Qin Decoction Preventing Colorectal Cancer Liver Metastasis

**DOI:** 10.3390/ph18071052

**Published:** 2025-07-17

**Authors:** Tiegang Li, Zheng Yan, Mingxuan Zhou, Wenyi Zhao, Fang Zhang, Silin Lv, Yufang Hou, Zifan Zeng, Liu Yang, Yixin Zhou, Zengni Zhu, Xinyi Ren, Min Yang

**Affiliations:** 1 State Key Laboratory of Digestive Health, Institute of Materia Medica, Chinese Academy of Medical Sciences and Peking Union Medical College, Beijing 100050, China; litiegang@imm.ac.cn (T.L.); yanzheng@imm.ac.cn (Z.Y.); zhoumx@imm.ac.cn (M.Z.); zhaowenyi@imm.ac.cn (W.Z.); zhangfang@imm.ac.cn (F.Z.); lvsilin@imm.ac.cn (S.L.); houyf@imm.ac.cn (Y.H.); zengzif@imm.ac.cn (Z.Z.); yangliu@imm.ac.cn (L.Y.); zhouyixin@imm.ac.cn (Y.Z.); zhuzengni@imm.ac.cn (Z.Z.); 23301997@muc.edu.cn (X.R.); 2 State Key Laboratory of Bioactive Substance and Function of Natural Medicines, Institute of Materia Medica, Chinese Academy of Medical Sciences and Peking Union Medical College, Beijing 100050, China; 3 School of Pharmacy, Minzu University of China, Beijing 100081, China

**Keywords:** Huang Qin Decoction, colorectal cancer, metabolomics, network pharmacology, single-cell RNA sequencing, molecular dynamics simulation

## Abstract

**Background:** Huang Qin Decoction (HQD) is a well-established Traditional Chinese Medicine (TCM) formulation recognized for its application in the treatment of colorectal cancer (CRC). However, the precise therapeutic mechanisms remain inadequately defined. **Methods:** This study integrates metabolomics from a mouse model and network pharmacology to screen potential targets and bio-active ingredients of HQD. The pharmacological activity of HQD for CRC was evidenced via single-cell RNA sequencing (scRNA-seq), molecular docking, and molecular dynamics simulations. Atomic force microscopy (AFM) assays and cellular experimental validation were used to confirm the relative mechanisms. **Results:** The metabolite profile undergoes significant alterations, with metabolic reprogramming evident during the malignant progression of CRC liver metastasis. Network pharmacology analysis identified that HQD regulates several metabolic pathways, including arginine biosynthesis, alanine, aspartate, and glutamate metabolism, nitrogen metabolism, phenylalanine metabolism, and linoleic acid metabolism, by targeting key proteins such as aspartate aminotransferase (GOT1), cytochrome P450 1A2 (CYP1A2), and carbonic anhydrase 2 (CA2). ScRNA-seq analysis indicated that HQD may enhance the functionality of cytotoxic T cells, thereby reversing the immunosuppressive microenvironment. Virtual verification revealed a strong binding affinity between the identified hub targets and active constituents of HQD, a finding subsequently corroborated by AFM assays. Cellular experiments confirmed that naringenin treatment inhibits the proliferation, migration, and invasion of CRC cells by downregulating GOT1 expression and disrupting glutamine metabolism. **Conclusions:** Computational prediction and *in vitro* validation reveal the active ingredients, potential targets, and molecular mechanisms of HQD against CRC liver metastasis, thereby providing a scientific foundation for the application of TCM in CRC treatment.

## 1. Introduction

Colorectal cancer (CRC) ranks as the third most prevalent cancer globally in terms of incidence and mortality, imposing a significant economic burden on households and society [1]. Liver metastasis represents the most common site of distant spread, with approximately 15–25% of patients developing hepatic metastasis at the time of initial diagnosis [2]. Despite advancements in chemotherapy, surgical techniques, and perioperative management, the prognosis for patients with CRC exhibiting liver metastasis remains dire, characterized by a 5-year relative overall survival rate of less than 10% [3]. Consequently, there is an urgent need for effective alternative therapies that are low in toxicity to reduce mortality, improve quality of life, and extend survival in patients with CRC [4].

Traditional Chinese Medicine (TCM) is a significant branch of complementary and alternative medicine (CAM) with a long-standing history of use in cancer treatment in China, noted for its cost-effectiveness and minimal side effects [5,6]. Huang Qin Decoction (HQD), a classical TCM formula documented in the Shang Han Lun by Zhang Zhongjing during the Han Dynasty, comprises *Scutellaria baicalensis* Georgi (Huangqin), *Paeonia lactiflora* Pall (Baishao), *Ziziphus jujuba* Mill (Dazao), and *Glycyrrhiza uralensis* Fisch (Gancao), and is widely utilized for alleviating various gastrointestinal symptoms. Modern phytochemical investigations have identified the major active components of HQD, including flavonoids (e.g., wogonin, baicalein, and oroxylin-A), flavonoid glycosides (e.g., wogonoside, baicalin, and oroxylin-A-glucoside), terpenoids, isoflavones, polysaccharides, and volatile oils [7]. These constituents exhibit diverse pharmacological properties, encompassing anti-inflammatory, analgesic, and immunomodulatory effects [8]. Accumulating evidence indicates that HQD may regulate amino acid homeostasis and the PI3K/AKT/mTOR signaling pathway, reduce colonic epithelial permeability, maintain colonic epithelial integrity, modulate mucosal immune activation, downregulate the effector phenotypes of Th1 and Th17 cells, and promote Th2 and Treg responses to alleviate colitis symptoms [8,9,10]. Notably, HQD exerts therapeutic effects on CRC by regulating the HIF-1 and mitogen-activated protein kinase signaling pathways [11]. Moreover, HQD has been shown to induce apoptosis via microbial butyrate-mediated PI3K/Akt inhibition, demonstrating anti-CRC activity [12]. Additionally, HQD may inhibit the initiation of colitis-associated carcinogenesis by modulating PAD4-dependent neutrophil extracellular traps [13]. PHY906, an oral powder derived from HQD, received orphan drug designation from the FDA in 2018. Phase I/II clinical studies reveal that PHY906 reduces gastrointestinal toxicity induced by the chemotherapeutic agent irinotecan by inhibiting NF-κB, COX-2, and inducible NO synthase pathways [14]. In clinical settings, HQD has been utilized in both the prevention and treatment of CRC [15], successfully alleviating intestinal symptoms in patients with advanced CRC [16]. However, due to the complexity of HQD’s composition and limitations in research methodologies, further investigation into the pharmacological mechanisms and molecular targets of HQD against CRC is warranted.

Network pharmacology represents a drug design methodology that integrates systems biology principles with network analysis techniques, accounting for connectivity patterns, redundancy mechanisms, and pleiotropic effects, which enables the construction of complex interaction networks to elucidate the regulatory principles of small molecules in a high-throughput manner, based on target molecules, biological functions, and bioactive compounds [17]. This approach aligns with the characteristics of TCM, which emphasize multi-component formulations, multi-target actions, and coordinated biological systems [18]. Consequently, network pharmacology has been effectively utilized to identify novel drugs, screen bioactive compounds, predict new disease targets, and elucidate pharmacological mechanisms and toxicity evaluations [18,19,20]. Wu et al. employed network pharmacology to demonstrate that quercetin, baicalin, and wogonoside in HQD exert therapeutic effects on ulcerative colitis by regulating targets such as TGS2, ESR1, and PPARG [21]. Additionally, it is well-recognized that tumor cells adapt their metabolic pathways to support and sustain malignant behaviors.

Metabolomics, as a comprehensive systems biology approach, is employed to meticulously monitor fluctuations in tumor metabolism and its responses to therapeutic interventions throughout disease progression [22]. This methodology provides insights into the intricate mechanisms underlying tumor pathogenesis and facilitates the optimization of treatment strategies. Using metabolomic analysis of plasma from patients with CRC liver metastasis, Costantini et al. [23] identified that levels of 3-hydroxybutyrate, cholesterol, phospholipids, triglycerides, and IL-6 can accurately categorize individuals based on their disease-free survival (DFS). Despite the established anti-inflammatory effects of HQD in ulcerative colitis and its widespread use in CRC prevention and treatment, a systematic analysis of HQD’s anti-CRC effects and the comprehensive mechanisms involved have not been thoroughly investigated. Thus, the integration of systems biology strategies, encompassing omics techniques and network pharmacology, has paved the way for exploring the multi-layered molecular mechanisms of HQD.

**Figure 1:**
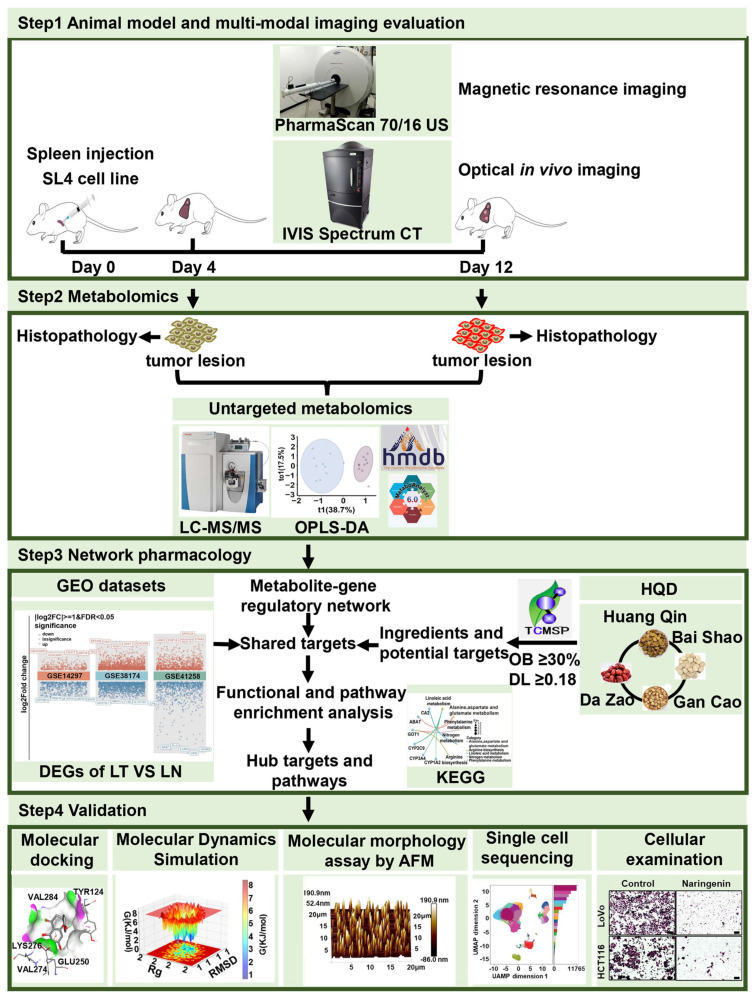
Detailed workflow to illustrate the molecular mechanism of HTD against the malignant progression of CRC liver metastasis. Abbreviations: HQD: Huang Qin Decoction, LN: liver normal, LT: liver tumor.

In this study, a mouse model of CRC liver metastasis was first established to analyze changes in metabolite profiles during malignant progression using metabolomics methodologies. Subsequently, the potential targets and pharmacological actions of HQD were explored through network pharmacology. By employing integrated approaches—including metabolomics, network pharmacology, scRNA-seq, bioinformatics analysis, molecular docking, molecular dynamics simulations, and experimental validation—this research elucidates the active components, key molecular targets, and potential mechanisms underlying HQD’s therapeutic effects against CRC for the first time. The objective of this study is to establish a robust scientific foundation to support the clinical application of HQD in CRC treatment, thereby advancing the development and application of TCM in the treatment and adjuvant management of CRC. A detailed flowchart is presented in Figure 1.

## 2. Results

### 2.1. In Vivo Multimodal Imaging to Monitor Liver Metastasis of CRC

**Figure 2:**
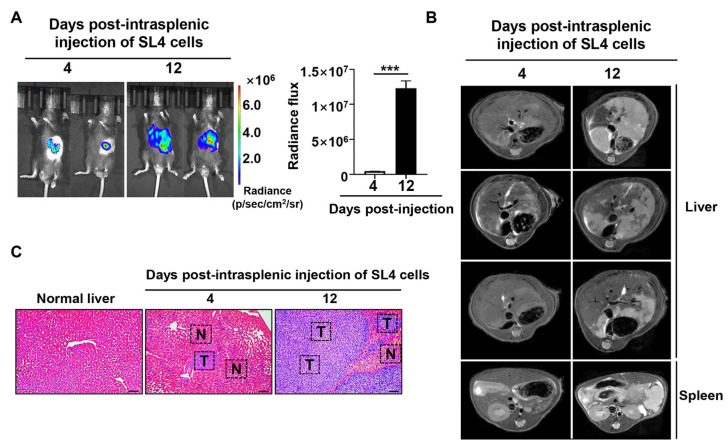
Assessment of tumor spread by multimodal imaging. (**A**) Longitudinal study using bioluminescence imaging to track tumor burden development. Quantitative analysis determined the total photon flux for each animal at specified times, *** *p* < 0.001. (**B**) *In vivo* T2-weighted MRI monitoring hepatic metastasis in CRC. (**C**) Representative images of H&E staining for mouse liver metastatic lesions. The black dashed line delineates the metastatic area from normal tissue. Scale bar = 100 μm, N: normal liver tissue, T: liver metastatic tumor foci.

To elucidate changes in global metabolite profiles, a liver metastasis CRC model was established in C57/6J mice by intrasplenically injecting SL4-luciferase murine colon cancer cells into the splenic capsule. Multimodal imaging was conducted at days 4 and 12 post-tumor inoculation. BLI results indicated that tumor cells primarily colonized the spleen, with no significant signs of tumor spread to the liver at day 4. In contrast, by day 12, both the intensity and extent of bioluminescent signals significantly increased in the liver, suggesting tumor dissemination to this organ (Figure 2A).

MRI further confirmed these results. T2-weighted images on day 4 revealed a slight high-intensity signal at the liver’s edge, indicative of early liver metastasis. Additionally, MRI showed a granular surface on the spleen, supporting the establishment of the tumor metastasis model. By day 12, the spleen appeared markedly enlarged, and a clear malignant lesion was visible in the liver. Subsequent histopathological evaluation of the liver showed a few dispersed metastatic foci at day 4, compared to normal liver tissue (Figure 2B). By day 12, liver metastases had become more pronounced, occupying over half of the field of view (Figure 2C). Overall, MRI provided a more accessible and sensitive approach for characterizing the mouse model of CRC liver metastasis compared to BLI.

### 2.2. Metabolism Rewiring in Malignant Progression of CRC Liver Metastases

**Figure 3:**
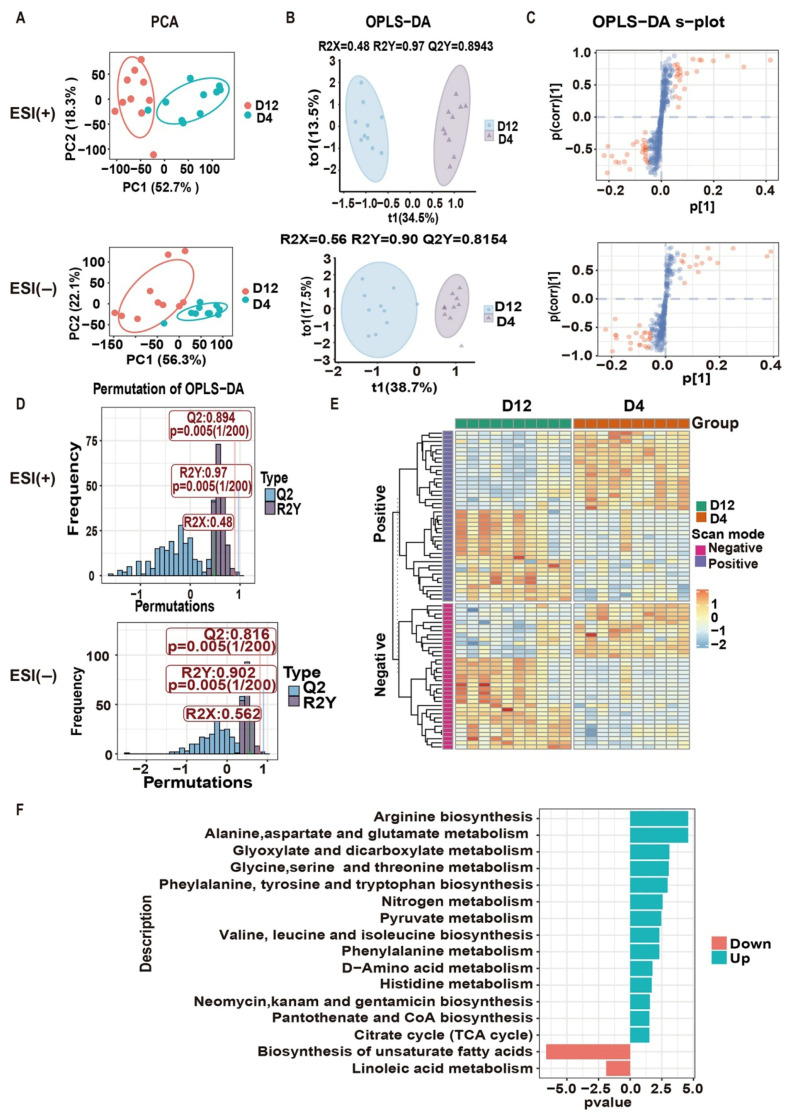
Untargeted metabolomics analysis. (**A**) PCA score plots in positive and negative ion modes. (**B**) OPLS-DA score plots in positive and negative ion modes. (**C**) S-plots from the OPLS-DA model, with red dots indicating metabolites with VIP values ≥1. (**D**) Permutation test results from the OPLS-DA model. (**E**) Heatmap of differential metabolites. (**F**) Pathway enrichment analysis based on differential metabolites using the MetaboAnalyst database.

To elucidate the shifts in metabolite profiles during cancer progression, a longitudinal untargeted metabolomics analysis was conducted on liver metastases at days 4 and 12 post-tumor cell inoculation. Initial PCA revealed substantial alterations in global metabolite features associated with tumor malignancy (Figure 3A). Subsequently, orthogonal partial least squares discriminant analysis (OPLS-DA) was employed to identify differential metabolites, showing distinct separation between the two groups in both positive and negative scan modes (Figure 3B). The model parameters were R2X = 0.48, R2Y = 0.97, Q2Y = 0.89 in positive mode and R2X = 0.56, R2Y = 0.90, Q2Y = 0.82 in negative mode. Additionally, a permutation test with 200 random permutations confirmed the models’ validity and absence of overfitting (Figure 3D). These results indicate that the OPLS-DA models based on metabolomic datasets from different tumor stages were robust and effective for identifying differential metabolites.

Differential metabolites were determined using VIP values (VIP ≥ 1) and *p*-values (*p* < 0.05) from the OPLS-DA model. The S-plot visually depicted each metabolite’s contribution to model classification, with red dots indicating metabolites with VIP values of 1 or greater (Figure 3C). Ultimately, 35 metabolites were significantly altered in the negative mode, and 41 in the positive mode between the D12 and D4 groups. Notably, the D12 group exhibited 44 upregulated and 32 downregulated metabolites compared to the D4 group, as illustrated in a heatmap (Figure 3E). Following this, KEGG pathway enrichment analysis was performed using the MetaboAnalyst 6.0 database to elucidate the role of these distinguished metabolites in malignant progression. A total of 16 significantly enriched metabolic pathways were identified, with 14 pathways strengthened and 2 weakened in the D12 group. These pathways were primarily associated with arginine biosynthesis, alanine, aspartate, and glutamate metabolism, nitrogen metabolism, and linoleic acid metabolism (Figure 3F). Collectively, untargeted metabolomics analysis indicates that metabolic reprogramming occurs during the malignant progression of CRC liver metastasis. Targeting these metabolic alterations may offer a promising strategy to mitigate the progression of CRC liver metastasis.

### 2.3. Elucidation of the Therapeutic Effects of HQD via Network Pharmacology

**Figure 4:**
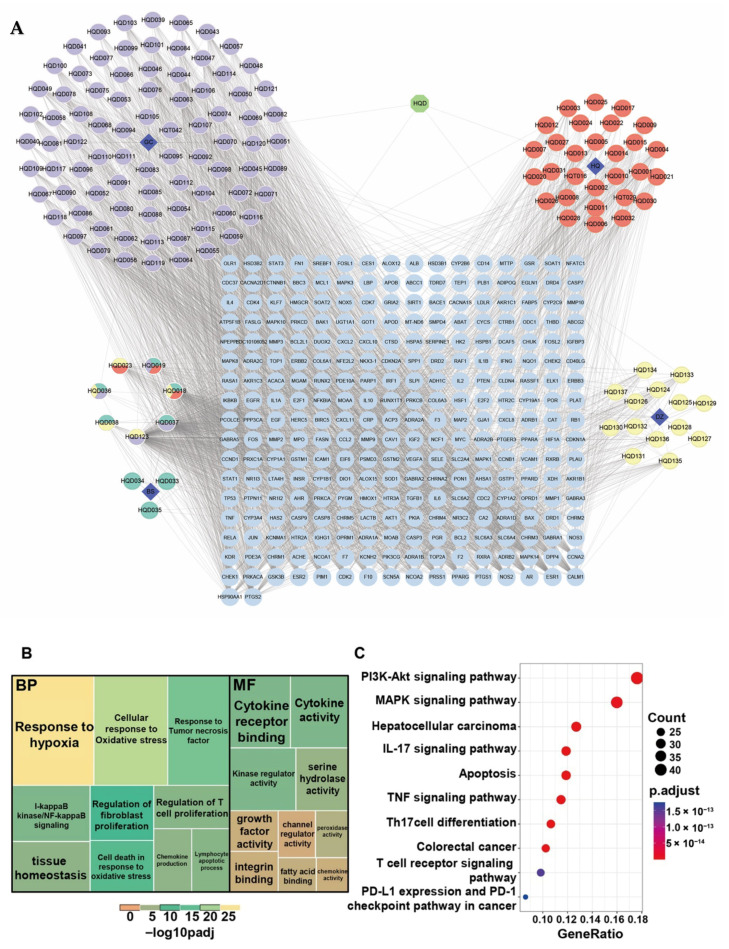
Pharmacological effects of HQD. (**A**) The “prescription-herbs-ingredients-targets” network. Green octagon nodes represent the prescription of HQD, blue diamond nodes indicate the herbs, and the colored ellipse nodes represent effective compounds from different herbal medicines. Light blue ellipse nodes arranged in a square denote therapeutic targets. (**B**) Treemap plot for GO enrichment analysis. (**C**) Bubble diagram for KEGG enrichment analysis.

To elucidate the underlying anti-tumor mechanisms of HQD, bioactive components were first gathered from the TCMSP database. A total of 137 active ingredients with suitable OB and DL were identified after removing duplicates. Additionally, 274 potential action targets were predicted, also excluding duplicates. A network depicting the interconnections among “prescription-herbs-ingredients-targets” was constructed, comprising 416 nodes and 2964 edges (Figure 4A). Notably, Gancao was associated with the highest number of active ingredients and potential therapeutic targets, while Baishao had the least.

Subsequently, GO and KEGG enrichment analyses were conducted to explore the potential functions of these therapeutic targets. The GO enrichment analysis revealed that response to hypoxia, oxidative stress, tumor necrosis factor signaling, tissue homeostasis, chemokine production, and regulation of T cell and fibroblast proliferation were significantly enriched in BP. In terms of MF, key activities included those related to cytokines, growth factors, channel regulation, chemokines, serine hydrolases, peroxidases, and kinase regulation, as well as integrin and fatty acid binding (Figure 4B). The KEGG enrichment analysis indicated that these genes may be involved in several signaling pathways, including PI3K-Akt, MAPK, TNF, T cell receptor, IL-17, PD1/PDL1, apoptosis, and pathways related to colorectal and hepatocellular cancers (Figure 4C).

### 2.4. Integrated Metabolomic, Transcriptomic, and Network Pharmacology Analysis to Unveil the Mechanism of HQD Against CRC Liver Metastasis

**Figure 5:**
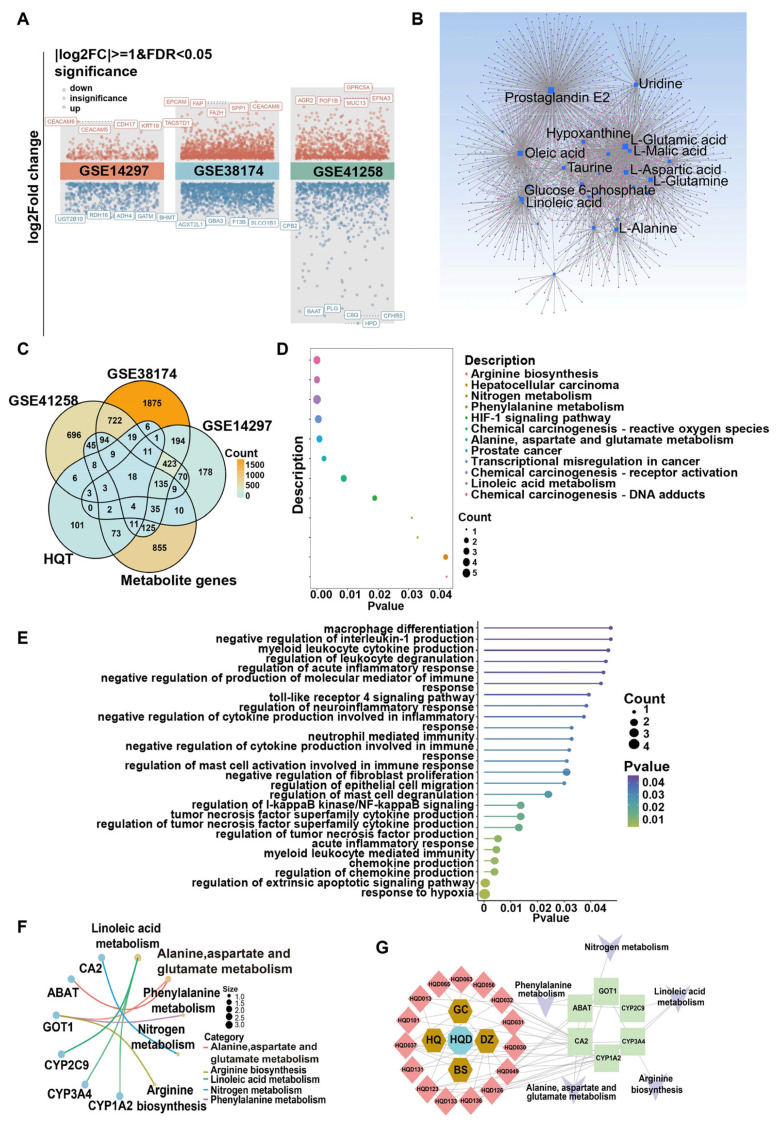
Mechanism of HQD in resisting malignant progression of CRC liver metastasis. (**A**) Volcano plot of DEGs from GEO datasets comparing normal liver tissue and CRC liver metastasis. (**B**) Interaction network of differential metabolites and genes, with colored dot nodes representing regulatory genes and square nodes indicating altered metabolites. Node size corresponds to degree value. (**C**) Venn diagram of HQD potential action targets for CRC liver metastasis. (**D**) KEGG enrichment analysis of hub targets. (**E**) GO enrichment analysis of hub targets. (**F**) Chordal graph of overlapping pathways between metabolic and KEGG enrichment results for HQD. (**G**) Network of “prescription-herbs-compounds-hub targets-core pathways”, with blue octagon nodes representing HQD prescriptions, yellow hexagon nodes indicating herbs, red diamond nodes for active ingredients, green rectangle nodes for key targets, and light green V-shaped nodes for significant pathways.

To identify key molecules that may intervene in the malignant progression of CRC liver metastasis, mRNA differential expression analysis was conducted using three GEO datasets containing normal liver tissues and samples from patients with CRC liver metastasis. The volcano plot (Figure 5A) indicates that in the GSE14297 dataset, 375 genes were upregulated and 720 downregulated compared to normal liver tissue; in the GSE38174 dataset, 1915 genes were elevated and 1767 reduced; and in the GSE41258 dataset, 1223 genes were overexpressed while 1047 were decreased. Figure 5B displays the interaction network of differential metabolites and genes, constructed using the online MetaboAnalyst 6.0 database. This analysis identified 1435 genes that may regulate the altered metabolites. A Venn diagram was then created from the differentially expressed genes (DEGs)of the GEO datasets, therapeutic targets of HQD, and genes associated with the differential metabolites, resulting in the identification of 18 hub targets for HQD’s treatment of CRC liver metastasis (Figure 5C).

To further elucidate the functions of these hub targets in CRC liver metastasis, KEGG and GO functional annotation analyses were performed. The KEGG enrichment analysis revealed significant associations with arginine biosynthesis, nitrogen metabolism, phenylalanine metabolism, alanine, aspartate, and glutamate metabolism, linoleic acid metabolism, as well as the HIF-1 signaling pathway, hepatocellular carcinoma, and prostate cancer (Figure 5D). GO term analysis indicated that these hub targets are mainly involved in macrophage differentiation, production of various chemokines and cytokines, immune response, epithelial cell migration, leukocyte and mast cell degranulation, hypoxia, and apoptosis (Figure 5E). Notably, five pathways were observed to overlap between the differential metabolite analysis and pathway enrichment results, specifically arginine biosynthesis, alanine, aspartate and glutamate metabolism, nitrogen metabolism, phenylalanine metabolism, and linoleic acid metabolism. Figure 5F illustrates these important pathways alongside six hub targets involved in them. Finally, a comprehensive network titled “prescription-herbs-compounds-hub targets-core pathways” was established to elucidate the material basis and molecular mechanisms by which HQD combats liver metastasis of CRC (Figure 5G). Collectively, these results suggest that HQD may exert its anti-tumor activity by regulating the immune microenvironment through multiple components and targets, thereby reshaping metabolic reprogramming.

### 2.5. Bulk Transcriptome Data Implying That HQD May Prevent Liver Metastasis of CRC by Regulating the Tumor Immune Microenvironment

**Figure 6:**
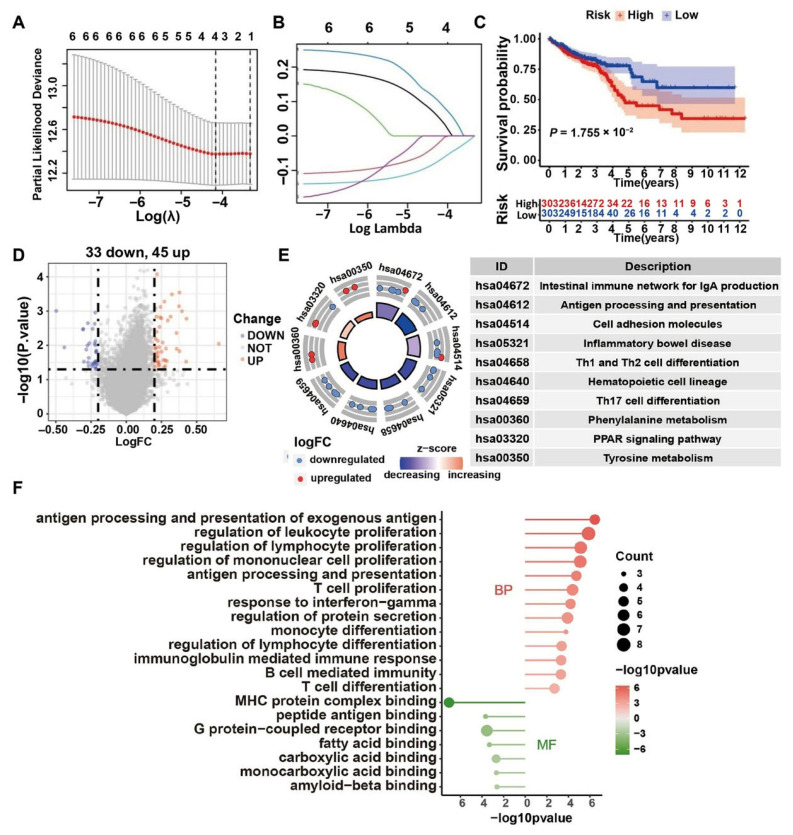
Lasso–Cox regression analysis for 18 potential therapeutic targets of HQD. (**A**) Selection of the tuning parameter (λ). (**B**) Profiles of LASSO coefficients. (**C**) KM curve of OS for low- and high-risk score subgroups. (**D**) Volcano plot illustrating the distribution of DEGs between the two subtypes. (**E**) Bar plot displaying GO enrichment analysis for DEGs. (**F**) Circular plot representing KEGG pathway enrichment analysis of DEGs.

Functional and pathway enrichment analyses of potential therapeutic targets of HQD indicate its capacity to exert anti-tumor effects through the regulation of the tumor immune microenvironment (TIME). A Lasso–Cox regression analysis was conducted to explore the relationship between six hub targets and immune profiles utilizing the CRC TCGA dataset (Figure 6A,B). Through 1000 iterations, four genes emerged as predictive factors for overall survival, with the risk score calculated using the following formula: risk score = (0.0462 × GOT1) + (−0.0139 × ABAT) + (0.0932 × CYP3A4) + (−0.0680 × CA2). Patients were subsequently stratified into high-risk (n = 303) and low-risk (n = 303) groups, using the median risk score as a cutoff. Kaplan–Meier analysis revealed that patients with CRC in the low-risk group exhibited significantly improved overall survival (OS) compared to their high-risk counterparts (Figure 6C). A total of 78 DEGs were identified, comprising 45 upregulated and 33 downregulated genes in the high-risk group relative to the low-risk group. The global expression changes of DEGs are illustrated in the volcano plot (Figure 6D). KEGG enrichment analysis indicated that DEGs predominantly participate in pathways related to intestinal immunity and inflammation, antigen processing and presentation, cell adhesion, as well as Th1, Th2, and Th17 cell differentiation, alongside phenylalanine and tyrosine metabolism (Figure 6E). GO term analysis of these DEGs demonstrated significant enrichment in processes such as antigen processing and presentation, lymphocyte proliferation and differentiation, and T/B cell proliferation and differentiation (Figure 6F).

**Figure 7:**
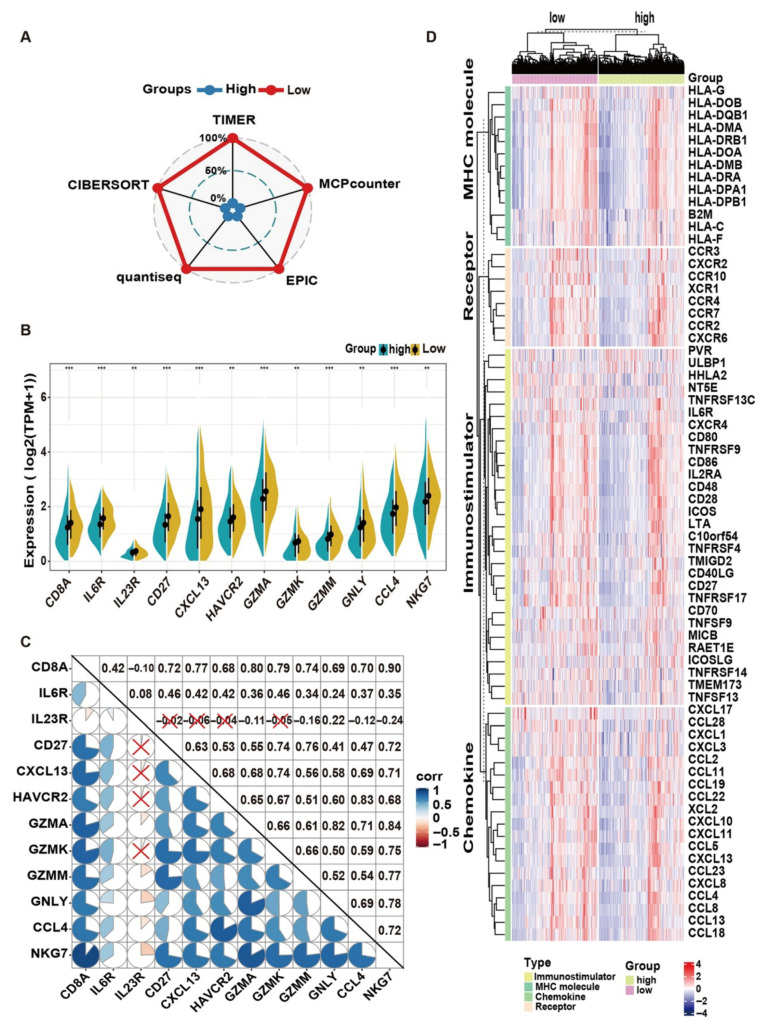
Immune profile analysis between high- and low-risk score cohorts. (**A**) Radar plot of relative infiltration levels of CD8+ T cells determined by multiple algorithms. (**B**) Abundance of marker genes related to the functionality of CD8+ T cells in low and high-risk groups. ** *p* < 0.01, *** *p* < 0.001. (**C**) Correlation plot depicting the expression levels of hallmark molecules associated with CD8+ T cell functionality. (**D**) Heatmap depicting immune-related molecular characteristics across the two clusters.

CD8+ tumor-infiltrating lymphocytes (TILs) play a pivotal role in tumor cell elimination through the secretion of cytokines and cytolytic molecules. Consequently, five immune infiltration algorithms were employed to estimate the relative infiltration levels of CD8+ T cells in tumor tissues. The radar plot (Figure 7A) indicates that the low-risk group exhibits higher infiltration levels of CD8+ T cells. Additionally, the expression levels of 12 gene signatures associated with CD8+ T cell functionality demonstrate significant differences between these two groups (Figure 7B). Correlation analysis reveals that most of these marker genes exhibit strong positive correlations, with the exception of IL23R (Figure 7C). Moreover, a notable abundance of MHC molecules, immunostimulators, and chemokines, along with their receptors, is evident in the low-risk group (Figure 7D). Based on these observations, HQD appears to facilitate the activation and infiltration of CD8+ T cells while reshaping the tumor immune microenvironment to combat liver metastasis in CRC.

### 2.6. Single-Cell Sequencing Analysis Confirms the Anti-Tumor Immune Activity of HQD

**Figure 8:**
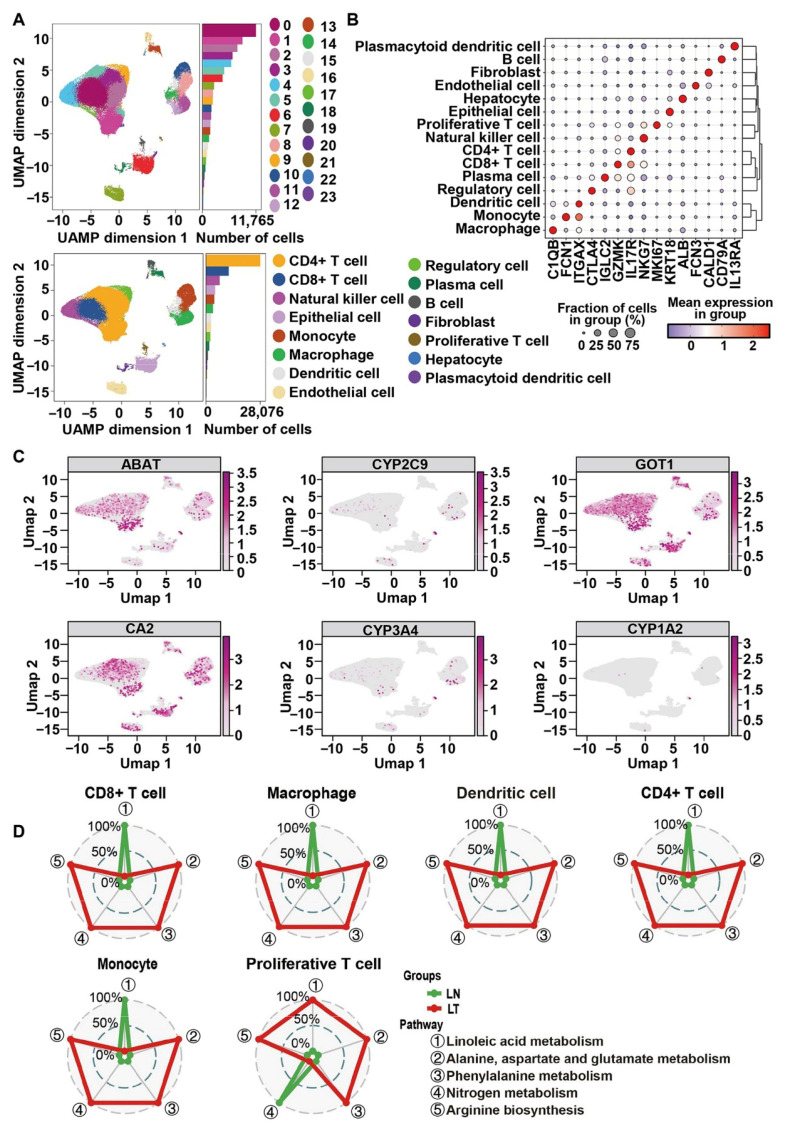
Single-cell transcriptome profiles to elucidate the potential mechanisms of HQD in combating liver metastasis of CRC. (**A**) UMAP plot for cell population identification. (**B**) Canonical cell markers across various clusters presented in a bubble heatmap, with dot size indicating the percentage of expressed cells and color representing their mean expression levels. (**C**) UMAP plot showing the distribution and expression levels of hub targets for HQD. (**D**) Radar graph exhibiting metabolic pathway activation across different cell types.

To elucidate the immunomodulatory effects of HQD, the previously published scRNA-seq dataset GSE231559 was re-analyzed to uncover differences in the immune microenvironment between liver metastases of CRC and adjacent non-cancerous samples. Following the preliminary quality control assessment and data filtration outlined above, a total of 71,319 cells were retained, encompassing 27,091 features. Based on the levels of highly variable genes, 24 distinct cell clusters were identified in the UMAP plot (Figure 8A,B). Utilizing the expression patterns of cell-specific markers, these 24 clusters were categorized into 15 different cell types.

Subsequently, the distribution and expression patterns of key targets associated with HQD in the context of malignant progression in CRC liver metastasis were analyzed. The findings reveal that these target genes predominantly localize to T cells, natural killer cells, macrophages, and epithelial cells, among others (Figure 8C). Additionally, the activity scores of five core metabolic pathways regulated by HQD across various cell types were calculated. The radar chart indicates that, with the exception of linoleic acid metabolism, the activities of other metabolic pathways are significantly enhanced in metastatic liver tissues from CRC compared to normal liver tissue. Notably, the activities related to nitrogen metabolism in proliferative T cells were markedly elevated in the pericarcinomatous tissue (Figure 8D). In summary, the metabolic characteristics evaluated at the single-cell level align with findings derived from metabolomic analyses.

**Figure 9:**
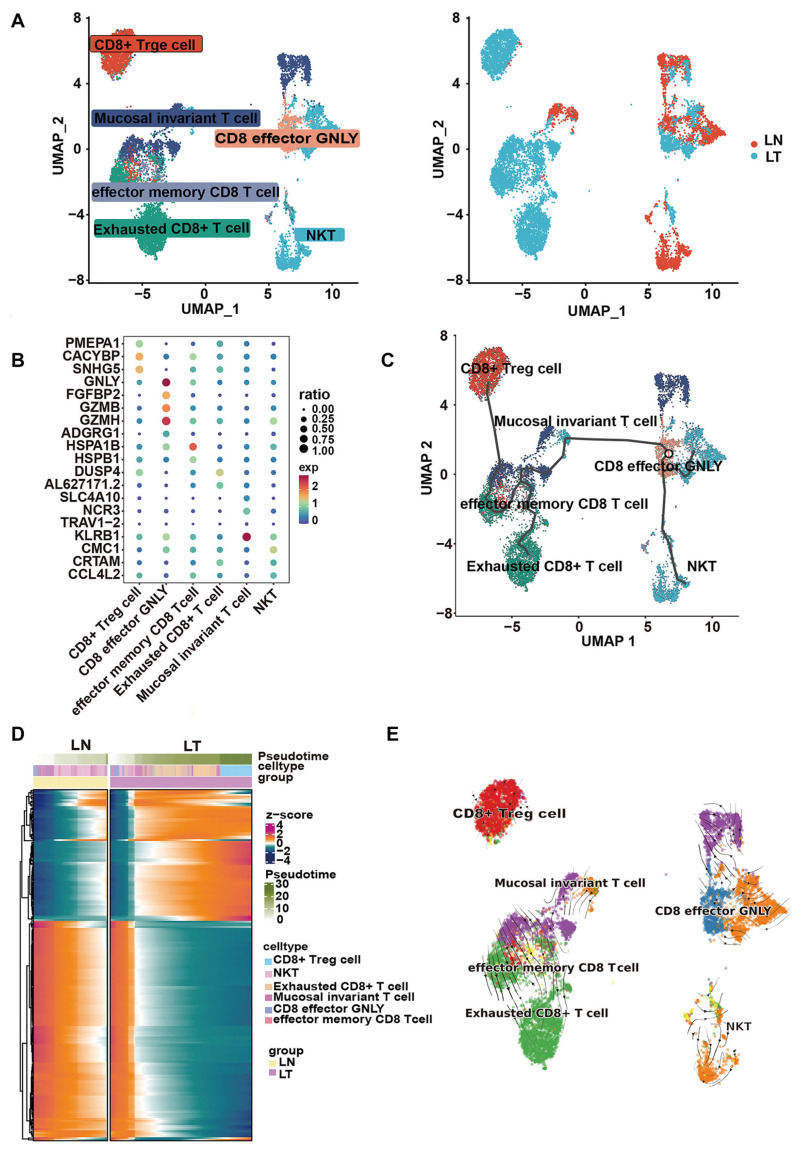
Pseudo-time differentiation trajectory analysis for CD8+ T lymphocytes. (**A**) UMAP plots display the subsets of CD8+ T cells (**left**) and their distribution in normal liver versus liver metastases (**right**) from CRC. (**B**) A bubble heatmap presents the expression patterns of marker genes specific to distinct cell types. (**C**) Trajectory plots illustrate the various clusters within CD8+ T cells. (**D**) A heatmap highlights the dynamic changes in gene expression along pseudo-time. (**E**) RNA velocity stream plots are utilized to infer lineage trajectories of CD8+ T cell subtypes.

To further elucidate the underlying mechanisms of HQD, a cell–cell communication analysis was conducted using the ‘CellChat V2.1.0’ package to comprehensively assess differences in the immune microenvironment between normal liver and liver metastases of CRC. The results indicate a higher estimated number of intercellular crosstalk events in normal liver, while the estimated strength of these interactions is greater at the tumor metastasis site (Figure S1A). Furthermore, the cell-to-cell communication network demonstrated significant alterations in both the quantity and strength of intercellular interactions, particularly among plasma cells, epithelial cells, CD8+ T cells, natural killer cells, CD4+ T cells, fibroblasts, and plasmacytoid dendritic cells (Figure S1B,C). These results suggest that the immune microenvironment in liver metastases from CRC is significantly altered compared to normal liver tissue, indicating that HQD may exert a therapeutic effect on tumors by modulating the TIME. Cytotoxic CD8+ T cells play a pivotal role in anti-tumor immunity, orchestrating immunogenic cell death through various mechanisms. Additionally, analysis of single-cell data revealed that the hub targets of HQD are predominantly located in CD8+ T cells. Notable alterations in the metabolic profiles and intercellular communication of CD8+ T cells were observed in metastatic liver lesions of CRC. Consequently, further analysis will focus on the subpopulations and pseudo-time differentiation trajectories of CD8+ T cells. Based on typical marker genes, CD8+ T cells were classified into six subgroups: CD8+ Treg cells, mucosal invariant T cells, CD8 effector GNLY cells, effector memory CD8 T cells, exhausted CD8+ T cells, and natural killer T (NKT) cells (Figure 9A). Further investigation revealed that NKT and CD8 effector GNLY cells are predominantly localized in normal liver tissues, while exhausted CD8+ T cells and CD8+ Treg cells are primarily found in liver metastases of CRC (Figure 9B). The inferred developmental trajectory suggests that effector CD8+ T cells are inclined to differentiate into exhausted-like and immunosuppressive phenotypes in tumor tissues (Figure 9C), with a heatmap depicting 203 distinct genes based on pseudo-time cell states (Figure 9D). Notably, RNA velocity analysis yielded intriguing insights into similarity lineage inference (Figure 9E). These results suggest that HQD may activate anti-tumor immune responses by inhibiting the differentiation of CD8+ T cells toward exhausted and immunosuppressive phenotypes, thereby combating the malignant progression of CRC liver metastasis.

### 2.7. Molecular Docking and Molecular Dynamic Simulation

**Figure 10:**
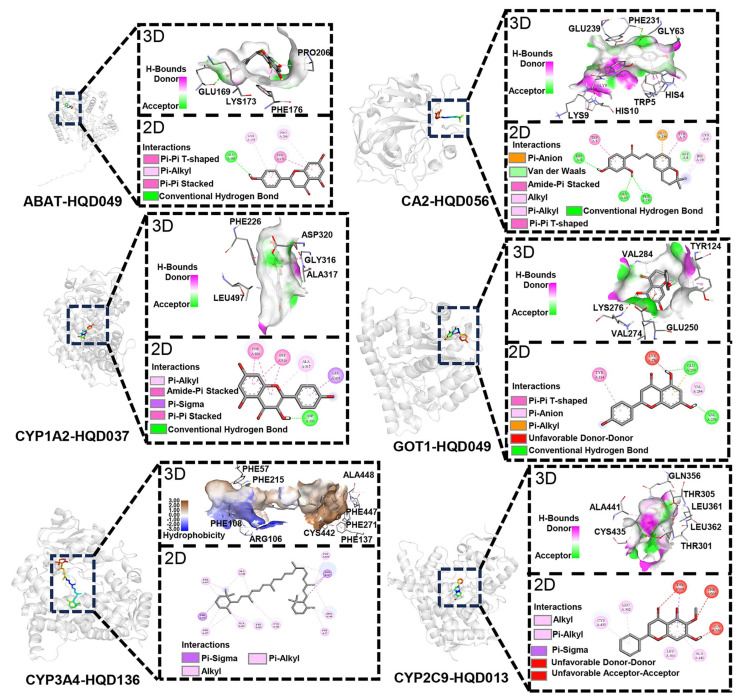
Schematic diagram of molecular interactions, featuring both 2D and 3D representations of the key components of HQD and their hub targets.

The key compounds and hub targets in HQD, as illustrated in Figure 5G, were selected for molecular docking to assess their potential for stable binding. The results are summarized in Supplementary File, Table S1. A binding energy lower than −5 kcal/mol indicates favorable binding activity, with lower binding energies reflecting stronger interactions between protein–ligand complexes. The binding scores for the molecules range from −6.5 to −10.0 kcal/mol, suggesting that all ligands and receptors exhibit favorable binding activities. Notably, the CYP1A2-HQD037 complex demonstrates the strongest binding affinity, as evidenced by the lowest binding energy. For each target, the complex with the lowest binding energy was selected for visualization (Figure 10).

**Figure 11:**
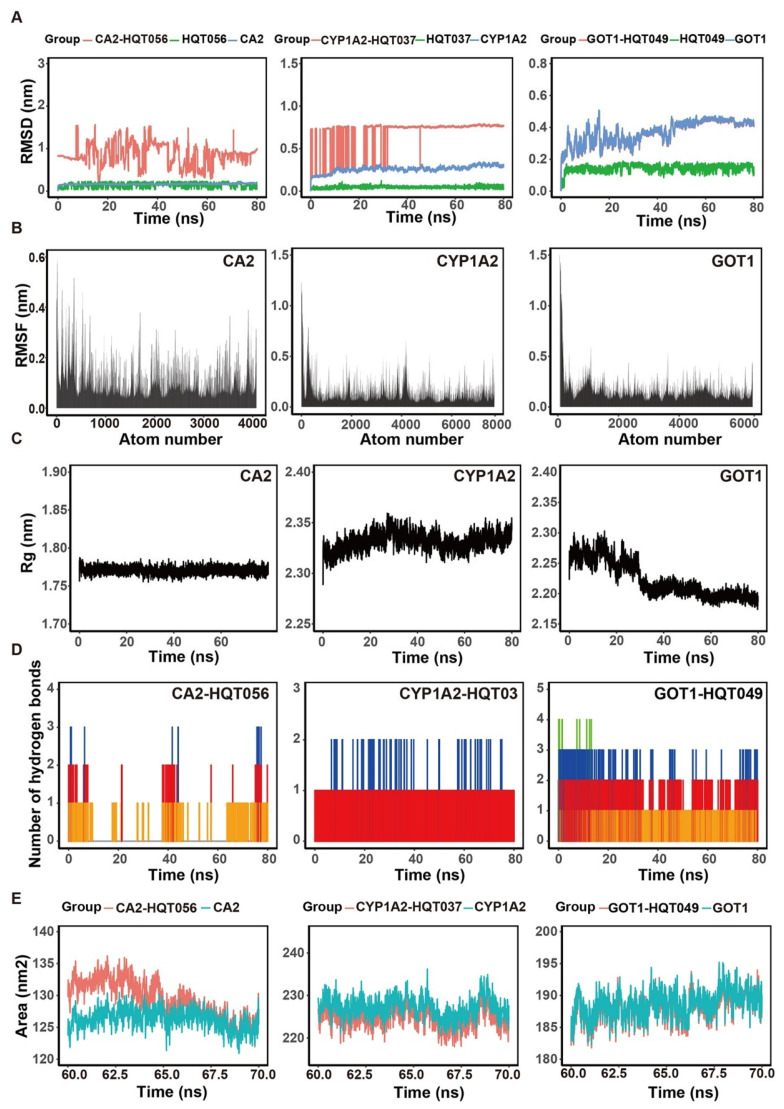
MD trajectory analysis for CA2-HQD056, CYP1A2-HQD037, and GOT1-HQD049, including (**A**) RMSD plots, (**B**) RMSF plots, (**C**) Rg plots. (**D**) Number of hydrogen bonds. (**E**) SASA plots.

Subsequently, to evaluate temporal stability, the complexes with the lowest binding energy from each significant pathway—CA2-HQD056, CYP1A2-HQD037, and GOT1-HQD049—were selected for 80 ns MD simulation. The RMSD analysis provides insights into structural conformation changes throughout the simulation. The fluctuation of all analyzed complexes remained below 1 nm, indicating conformation stabilization (Figure 11A). The RMSF reflects the movement amplitude of amino acid residues; results show that GOT1 and CYP1A2 exhibit greater residue flexibility compared to CA2 (Figure 11B). Rg offers insights into the compactness of the binding and the degree of protein folding. An Rg value around 2 nm suggests that the system is dense and tightly packed (Figure 11C). Hydrogen bonding, a key non-covalent interaction, contributes to the stability of the complex. During the MD simulation, CA2-HQD056 maintained 0–3 hydrogen bonds, CYP1A2-HQD037 had 1–2, while GOT1-HQD049 exhibited 1–4 hydrogen bonds (Figure 11D). The SASA value illustrates the spatial arrangement of protein residues in the solvent environment. Results indicate that the complex and the protein exhibit similar SASA values, suggesting that the volume of the protein does not significantly expand upon ligand binding (Figure 11E).

**Figure 12:**
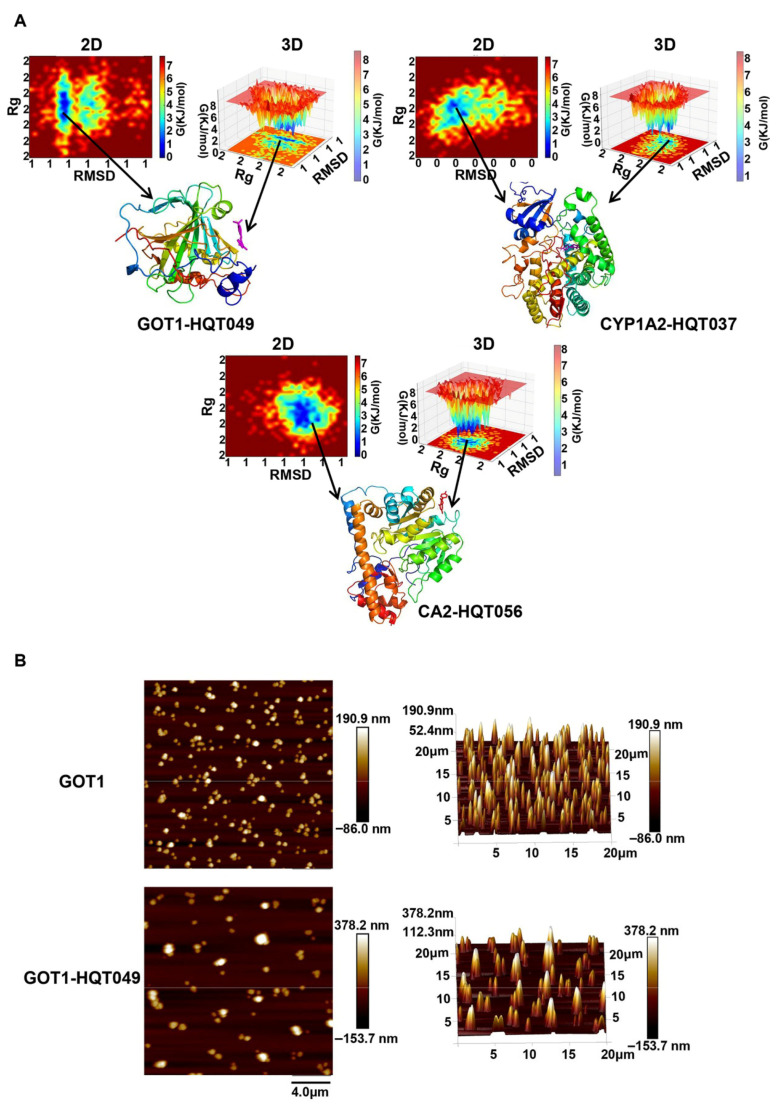
Validation of the findings in MD simulation. (**A**) The 2D and 3D Gibbs free energy landscapes for CA2-HQD056, CYP1A2-HQD037, and GOT1-HQD049 are plotted against RMSD and Rg. The color scale is based on kcal/mol, where darker blue shades represent lower energy states, indicating more stable conformations. (**B**) 2D and 3D surface morphology images of the GOT1 protein and the GOT1-HQD049 complex obtained through AFM analysis.

Furthermore, RMSD and Rg were employed to define the Gibbs free energy landscape, which describes the distribution of binding free energy and captures the steady-state structures of the complexes during MD simulation. Figure 12A presents the 2D and 3D free energy topography maps and the optimal conformations of the complexes based on the 60–70 ns MD simulation trajectory, with deep blue colors indicating the lowest energy states. Additionally, binding free energy measurements provide further evidence for the stability of the complexes, as shown in Supplementary File, Table S2. The average total binding free energy in MMGBSA for CA2-HQD056, CYP1A2-HQD037, and GOT1-HQD049 is (−6.17 ± 3.66), (−25.57 ± 0.84), and (−15.41 ± 1.85) kcal/mol, respectively. Similarly, the average total binding free energy in MMPBSA for these complexes is (−7.02 ± 3.67), (−16.87 ± 2.36), and (−10.81 ± 2.01) kcal/mol, respectively. These results reveal that the key ingredients and hub targets can stably bind to exert pharmacological effects.

### 2.8. Molecular Surface Topography Analysis by AFM

To further confirm the binding of active ingredients and key targets, AFM analysis was conducted to visually display intermolecular interactions (Figure 12B). Under identical protein concentration and testing conditions, GOT1 protein particles appeared small and evenly distributed on the mica surface. In contrast, the GOT1–naringenin complexes exhibited aggregation and increased size. The maximum height of the protein was measured at 190.9 nm, which increased to 378.2 nm upon ligand binding, as illustrated in the 3D topology images. Overall, AFM images reaffirmed that naringenin can stably bind to GOT1 protein, influencing its function and contributing to anti-tumor effects [24,25,26].

### 2.9. Validation of the Anti-CRC Effect of Naringenin In Vitro

**Figure 13:**
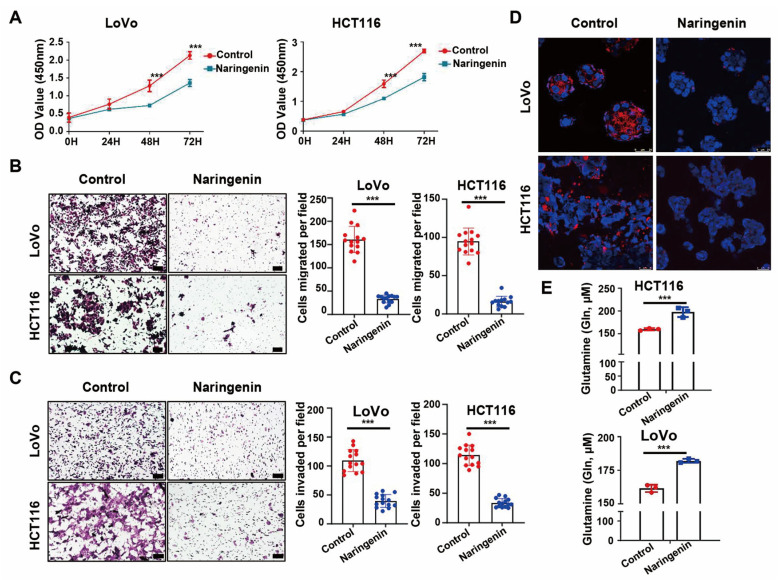
Naringenin attenuates biological behaviors, decreases GOT1 expression, and disrupts glutamine metabolism in malignant colon cancer cells. (**A**) The CCK-8 assay was utilized to evaluate cell viability. Transwell assays quantified the number of migrated (**B**) and invaded (**C**) cells (×10). Immunofluorescence assays detected GOT1 expression (**D**) (×60), while ELISA assays measured glutamine levels in cell supernatants (**E**). *** *p* < 0.001.

To evaluate whether the active ingredients in HQD can inhibit tumor growth, two malignant colon cancer cell lines, LoVo and HCT116, were cultured to assess the bioactivities of naringenin regarding anti-proliferation, effects on malignant behaviors, GOT1 expression, and glutamine metabolism. The results demonstrated that naringenin significantly reduced proliferation, migration, and invasion *in vitro* (Figure 13A–C). Additionally, naringenin treatment led to decreased protein expression of GOT1 and reduced glutamine uptake (Figure 13D,E).

## 3. Discussion

Based on empirically accumulated knowledge, TCM has a long-standing history in China as a clinical adjuvant for cancer treatment, achieving notable curative outcomes [27]. HQD, a classic and renowned TCM prescription, has been utilized in China for over 1800 years, recognized for its efficacy in clearing heat, alleviating dysentery, and relieving pain through regulation of the middle-jiao. Clinically, it is primarily employed to treat gastrointestinal dysfunctions such as diarrhea, nausea, and vomiting [28]. Previous studies indicate that HQD ameliorates ulcerative colitis through various mechanisms, including the regulation of fatty acid metabolism to promote M2 macrophage polarization via the FFAR1/FFAR4-AMPK-PPARα pathway, modulation of gut microbiota, and amino acid metabolism, as well as activation of the mTOR signaling pathway to restore epithelial barrier function [29,30]. Additional research has demonstrated that HQD can improve gut dysbiosis and induce apoptosis in CRC mouse tumor cells through microbial butyrate-mediated inhibition of the PI3K/Akt pathway [12]. Data from Phase I/II clinical trials released by the National Institutes of Health (NIH) in the United States confirm that HQD reduces the intestinal side effects of chemotherapy and enhances chemotherapeutic efficacy in patients with CRC [14,31]. Furthermore, HQD has been shown to synergize with immune checkpoint inhibitors, chemotherapy, and targeted therapies in treating hepatocellular carcinoma, thereby maximizing therapeutic benefits, minimizing adverse effects, and improving patient survival by modifying the tumor microenvironment [32,33,34]. Despite its proven efficacy, the complexity of HQD’s compound composition and the multifaceted mechanisms of action involving multiple components, pathways, and targets complicate the elucidation of its precise anti-CRC effects. To address these challenges, this study aims to predict and validate the potential active components and mechanisms of HQD for CRC treatment using metabolomics, network pharmacology, scRNA-seq, molecular docking, molecular dynamics simulation, and experimental validation.

Reprogramming cellular metabolism has emerged as a hallmark of cancer, playing a critical role in tumor initiation, progression, metastasis, therapy resistance, and immune evasion [35]. Consequently, targeting these aberrant metabolic pathways presents an innovative therapeutic strategy with the potential to overcome treatment resistance and improve therapeutic outcomes [36]. To investigate metabolic profile alterations during CRC liver metastasis, a murine model of colon cancer liver metastasis was established, as detailed in our previous research [37]. Non-targeted metabolomics analysis revealed significant changes in the metabolic profiles of hepatic tumor nodules corresponding with advanced metastatic behavior. Notably, pathways involved in arginine biosynthesis, alanine, aspartate, and glutamate metabolism, nitrogen metabolism, and phenylalanine metabolism were significantly enhanced, while linoleic acid metabolism and biosynthesis of unsaturated fatty acids were dramatically downregulated. It has been reported that arginine supplementation inhibits tumor growth and crypt cell hyperproliferation during the initiation phase of tumorigenesis, but may stimulate tumor growth during the promotion stage in CRC [38]. Global metabolomics analysis indicated activation of alanine, aspartate, and glutamate metabolic pathways in patients with CRC, suggesting that key enzymes in these pathways could be promising targets for cancer therapy [39,40]. Nitric oxide (NO), a signaling molecule in nitrogen metabolism, mediates Wnt/β-catenin and extracellular-signal-regulated kinase (ERK) pathways in CRC. Thus, NO and NO synthases (NOS) are closely linked to cancer initiation, metastasis, inflammation, and resistance to chemotherapy and radiation [41]. Elevated phenylalanine levels have been associated with systemic inflammation in patients with CRC, and high phenylalanine levels correlate with decreased survival in a 120-month follow-up study [42]. Linoleic acid promotes cellular quiescence by enhancing the expression of miR-494, leading to cancer cell dormancy [43]. A recent meta-analysis involving 54 patients with CRC suggested that highly unsaturated fatty acids may be associated with a reduced risk of CRC [44]. Overall, metabolomics has elucidated differential metabolite profiles between the early and late stages of liver metastasis in colon cancer, highlighting metabolic pathways relevant to the therapeutic effects of HQD.

Network pharmacology, utilizing integrated databases and advanced analytical strategies, has gained prominence in the past decade for exploring the relationships between TCM and diseases at the molecular and systemic levels. In this study, this approach was employed to construct a compound–target–pathway network to investigate the anti-CRC effects of HQD. Enrichment analysis of target genes related to the active ingredients indicates that HQD can regulate tumor cell growth and enhance the tumor immune microenvironment. Previous evidence suggests that HQD reduces levels of inflammatory factors such as IL-1β, IL-6, and TNF-α while targeting the WNT pathway to inhibit CRC progression [45]. Additionally, HQD decreases the levels of Treg cells, thereby maintaining intestinal stability and reducing the risk of colon cancer [46]. The present study identified six hub targets—GOT1, CYP2C9, CYP34, CYP1A2, CA2, and ABAT—that are involved in five core pathways related to HQD’s effects on malignant progression in colorectal cancer liver metastases, integrating transcriptomic and metabolomic analyses. GOT1 (Glutamate oxaloacetate transaminase 1) plays a pivotal role in metabolizing glutamine to produce nicotinamide adenine dinucleotide phosphate (NADP+) and nicotinamide adenine dinucleotide (NAD+), thereby supporting redox balance and tumor growth [47]. GOT1 is overexpressed in many CRC cell lines, and its inhibition sensitizes CRC cells to 5-fluorouracil (5-FU) by compromising their defenses against 5-FU-induced reactive oxygen species (ROS) [48]. CYP2C9, CYP34, and CYP1A2 are members of the cytochrome P450 family, involved in the metabolism of various endogenous and exogenous substrates. Studies have shown that the levels of low-spin forms of cytochrome P450 in liver metastases and adjacent tissues are lower than in conventionally normal liver tissues [49]. CA2 (Carbonic anhydrase 2) regulates ion transport and pH balance and has been shown to suppress CRC cell growth significantly, both *in vitro* and *in vivo*, when overexpressed [50]. ABAT (4-aminobutyrate aminotransferase) catalyzes the conversion of γ-aminobutyrate (GABA) and L-β-aminoisobutyrate to succinate semialdehyde and methylmalonate semialdehyde, respectively, and exhibits higher expression in CRC tissues [51]. This study uniquely combines network pharmacology and bioinformatics methods to elucidate the specific mechanisms through which HQD treats CRC. The findings support several of our predicted results, providing a theoretical foundation for understanding the complex therapeutic mechanisms of HQD.

To further elucidate the immunomodulatory effects of HQD, the TCGA dataset for CRC was analyzed, leading to the construction of a Lasso–Cox regression model based on the six identified hub targets. Patients were stratified into high-risk and low-risk groups according to the derived risk scores. Results indicated a significant reduction in the infiltration levels of CD8+ T cells, as well as decreased expression of various cytokines and tumor-killing receptors in the high-risk cohort. Additionally, analysis of single-cell sequencing data from the GEO database revealed that the hub targets associated with HQD treatment were predominantly localized within various T cell subpopulations. Single-cell metabolic activity analysis demonstrated that, relative to normal liver tissue, the metabolic activity of most core pathways—excluding linoleic acid metabolism—was significantly enhanced in tumor-killing cells, including CD8+ T cells, CD4+ T cells, macrophages, dendritic cells, monocytes, and proliferative T cells, aligning with findings from metabolomics analyses. Given the critical role of cytotoxic CD8+ T cells in eradicating malignant cells through the secretion of cytotoxic granules, these cells were further subdivided into subclusters for pseudo-time and RNA velocity analysis. The results indicated that CD8+ T lymphocytes in the liver metastatic foci of CRC primarily exhibited exhausted and regulated states, lacking tumor-killing capabilities compared to their counterparts in normal liver tissue. These findings suggest that HQD may target key proteins such as GOT1, ABAT, and CYP1A2 to modulate pathways related to arginine biosynthesis, alanine, aspartate, and glutamate metabolism, nitrogen metabolism, phenylalanine metabolism, and linoleic acid metabolism. This modulation may inhibit the transition of CD8+ T cells to exhausted and regulatory phenotypes, thereby resisting the malignant progression of CRC liver metastasis.

Subsequent molecular docking analyses confirmed the stability of the complexes formed between the hub targets and the active ingredients in HQD, with docking binding free energies consistently below −5 kcal/mol, indicating stable conformations. Molecular dynamics simulations further corroborated these findings through comprehensive analyses of parameters such as RMSD, RMSF, hydrogen bonding, SASA, and MMGB/PBSA, demonstrating that these active compounds exhibit favorable binding activity to their targets, thus contributing to the therapeutic effects of HQD in CRC. Notably, AFM assays indicated that the active ingredient naringenin significantly alters the morphology of the GOT1 protein, suggesting stable binding that modulates its function, thereby achieving therapeutic effects against the disease. These observations validate results obtained from both molecular docking and molecular dynamics simulations.

Cellular experiments revealed that naringenin significantly inhibits the proliferative, invasive, and migratory capabilities of CRC cells. A marked down-regulation of intracellular GOT1 protein expression levels was observed following naringenin administration, which corresponded with repression of glutamine metabolism in CRC cells. Previous studies have demonstrated that naringenin interacts with estrogen receptors, promotes apoptosis, inhibits enzymes associated with cell survival and proliferation, and decreases the expression of cyclin-dependent kinases in CRC cells, indicating its potential as a therapeutic agent for CRC [52]. Furthermore, naringenin is suggested to inhibit CRC proliferation, possibly through the AMPK pathway, regulating mitochondrial function and inducing apoptosis [53]. However, the metabolism-related mechanisms underlying the beneficial effects of naringenin on CRC cells remain unclear. Emerging research emphasizes that glutamine metabolism plays a pivotal role in promoting cancer cell proliferation by maintaining redox homeostasis and serving as a precursor for biomass synthesis [54]. Therefore, targeting the inhibition of GOT1 to disrupt tumor glutamine-mediated metabolic reprogramming represents an important strategy for screening promising anti-tumor drug candidates. Collectively, these results suggest that naringenin targets the GOT1 protein to inhibit the glutamine metabolic pathway, thereby suppressing tumor development in CRC.

This study presents several limitations. First, an untargeted metabolomics approach was employed to examine metabolic profile changes during CRC liver metastasis; however, the key differential metabolic pathways identified lack targeted metabolomic and experimental validation. Second, bioactive compound data were sourced from literature and databases, necessitating further exploration of the active compounds of HQD using LC/MS technology in future studies. Additionally, only some key components, core targets, and metabolic pathways of HQD for CRC treatment were confirmed through AFM analysis and cellular experiments; more thorough validation would enhance the characterization of the multi-target, multi-component actions of HQD. Future comprehensive studies, incorporating animal experiments, are essential to elucidate the therapeutic targets and pharmacological mechanisms of HQD.

## 4. Materials and Methods

### 4.1. Development of CRC Liver Metastasis Animal Tumor Model and Multimodal In Vivo Imaging Evaluation

Eight- to ten-week-old specific pathogen-free C57/6J male mice were sourced from Beijing Vital River Laboratory Animal Technology (Beijing, China). Following a week of acclimatization, the mice were randomly assigned to two groups (n = 10 per group): the day 4 group (D4) and the day 12 group (D12). A total of 1 × 10^6^ SL4-luciferase murine colon cancer cells were injected intrasplenically to establish a mouse model of CRC liver metastasis. Bioluminescence imaging (BLI) and small animal magnetic resonance imaging (MRI) were performed on days 4 and 12 post-operation to assess tumor metastasis. Mice were euthanized on day 4 and day 12 according to the experimental design, and tumor tissues from liver metastases were harvested and immediately stored at −80 °C for subsequent pathological and metabolomic analyses. Liver tissues with tumor invasion were fixed in 4% paraformaldehyde and sectioned into 5 μm-thick slices for histological assessment. The specific mouse modeling process and imaging procedures are described in detail in the Supplementary Materials and Methods. All experimental protocols were approved by the ethics committee for Animal Experiments at the Institute of Materia Medica, Chinese Academy of Medical Sciences, and Peking Union Medical College.

### 4.2. Untargeted Metabolomics Analysis

Frozen colorectal liver metastasis tumors from days 4 and 12 post-surgery were cut into small pieces, weighed to 50 mg, and placed into 2 mL Eppendorf tubes to prepare tissue homogenate for detection. Metabolomic analysis was conducted using a Thermo Fischer Dionex UltiMate 3000 ultra-high-performance liquid chromatography (UHPLC) system, coupled to a Q-Orbitrap mass spectrometer (Q Exactive, Thermo Scientific, Bremen, Germany) with an HESI probe. Data acquisition was analyzed using Xcalibur 2.3 software. Chromatographic separation was performed on a Waters Acquity CSH C18 column (1.7 μm, 2.1 mm × 100 mm, Waters, Milford, MA, USA ) with the column oven maintained at 30 °C. Detailed conditions for chromatographic and mass spectrometric analysis, along with the data processing procedures, are provided in the Supplementary Materials and Methods.

### 4.3. Network Pharmacology Research on the Anti-Tumor Effect and Mechanism of HQD

#### 4.3.1. Active Components Collection and Potential Targets Prediction of HQD

The Traditional Chinese Medicine Systems Pharmacology Database and Analysis Platform (TCMSP, https://old.tcmsp-e.com/tcmsp.php (accessed on 1 January 2024)) was utilized to filter active components and predict potential targets of HQD [55]. Based on pharmacokinetic properties (absorption, distribution, metabolism, and excretion, ADME), only constituents with oral bioavailability (OB) ≥ 30% and drug-likeness (DL) ≥ 0.18 were selected as bioactive compounds. Subsequently, the gene and protein target information was converted to gene symbol names using the UniProt database (https://www.uniprot.org/ (accessed on 1 January 2024)).

#### 4.3.2. Identification of Therapeutic Targets Related to CRC Liver Metastasis

Three CRC liver metastasis mRNA gene expression datasets—GSE41258, GSE38174, and GSE14297—were retrieved from the Gene Expression Omnibus (GEO) database (https://www.ncbi.nlm.nih.gov/geo/ (accessed on 3 January 2024)), which include metastatic lesions and normal tissues adjacent to the cancer. Then, the ‘limma V3.62.2’ package was used to screen differentially expressed genes (DEGs). Detailed information on the datasets and the processing procedures can be found in the Supplementary Materials and Methods.

#### 4.3.3. Integrated Analysis of Metabolome, Transcriptome, and Network Pharmacology to Screen the Key Targets for HQD Against CRC Liver Metastasis

To identify potential therapeutic targets of HQD, DEGs from normal liver versus CRC liver metastasis and genes regulating altered metabolites from metabolomics analysis were intersected. A Venn diagram was generated using the ‘ggVennDiagram V1.5.4’ R package to illustrate these key targets.

#### 4.3.4. Functional and Pathway Enrichment Analyses

To further elucidate the therapeutic effects of HQD and its potential mechanisms in treating CRC liver metastasis, Gene Ontology (GO) and Kyoto Encyclopedia of Genes and Genomes (KEGG) analyses were conducted using the ‘clusterProfiler V4.14.6’ R package.

#### 4.3.5. Hub Targets and Core Ingredients Screening as Well as the Construction of Prescription–Herbs–Compounds–Targets–Pathways Network

Overlapping pathways between the KEGG pathways enriched for HQD targets and those enriched for differential metabolites from untargeted metabolomics were considered potential regulatory mechanisms of HQD in treating CRC liver metastasis. Genes within these overlapping pathways were designated as hub targets, and the ingredients in HQD that can modulate these hub targets were identified as core ingredients. Finally, a network comprising prescription herbs, compounds, targets, and pathways was constructed based on the protein–protein interaction (PPI) network using Cytoscape 3.10.1 software [56].

### 4.4. Verification of the Mechanism of HQD in Hampering CRC Liver Metastasis

#### 4.4.1. Molecular Docking

The 3D protein crystal structures of hub targets were downloaded in PDB format from the RCSB PDB database (https://www.rcsb.org/), while the 2D structures of core compounds were extracted from the PubChem database (https://pubchem.ncbi.nlm.nih.gov/ (accessed on 25 January 2024)) and saved in SDF format. Prior to molecular docking, the small molecular ligand SDF files underwent energy minimization using the full minimization method and were converted to MOL2 format via Discovery Studio 2023 software V23.1.100.23209. Docking simulations were conducted with AMDock software v1.6.2 [57]. The docking grid box was constructed with the AutoLigand tool [58] (without co-crystallized complex) or center on the ligand (with co-crystallized complex). The force field is the Vina force field.

#### 4.4.2. Molecular Dynamics (MD) Simulation

The receptor–ligand complexes with the lowest binding energies identified through molecular docking were selected for 80 ns molecular dynamics simulations using GROMACS 2022 [59]. The specific process is detailed in the Supplementary Materials and Methods. To confirm the stability of the complex, parameters such as root mean square deviation (RMSD), root mean square fluctuation (RMSF), radius of gyration (Rg), solvent accessible surface area (SASA), and the number of hydrogen bonds (H-bonds) were calculated using molecular dynamics trajectory files. Meanwhile, binding free energy using Molecular Mechanics with Generalized Born and Surface Area solvation (MM/GBSA) and Molecular Mechanics Poisson–Boltzmann Surface Area (MM/PBSA) was also evaluated via the gmx_MMPBSA tool V1.6.3 [60]. Finally, the Gibbs free energy landscape (FEL) was plotted using RMSD and Rg data to illustrate the conformation energy distribution of the complex during molecular dynamics.

#### 4.4.3. Molecular Morphology Assay by Atomic Force Microscope (AFM)

Recombinant human aspartate aminotransferase (GOT1) protein (10 μg) was procured from MedChemExpress (Shanghai, China) and dissolved in 50 μL water, then diluted to a 200 μg/mL solution. Naringenin powder, obtained from Sigma-Aldrich with 98% purity, was precisely weighed at 10 mg and dissolved in 50 mL of a 20% ethanol solution to yield a 200 μg/mL solution. The protein and ligand solutions were mixed in a 1:1 ratio and incubated in a water bath at 37 °C for 30 min to form a 100 μg/mL complex solution. The protein solution was also diluted one-fold for determination. Subsequently, 10 μL of both complex and protein solutions were quickly deposited onto the surface of a mica sheet. Samples were freeze-dried for 6 h to crystallize and remove water prior to analysis. Morphological evaluation of the complex and protein was conducted using a Dimension ICON AFM (Bruker, Santa Barbara, CA, USA) in tapping mode, with a scan rate of 1.0 Hz and a scan size of 20 × 20 μm. Captured images were processed using NanoScope Analysis 1.90 software.

#### 4.4.4. Bioinformatics Analysis for TCGA Datasets of CRC

##### Construction of Risk Score Model

mRNA expression profiles and clinicopathological annotation information of patients with CRC were retrieved from the TCGA database (https://www.cancer.gov/ccg/research/genome-sequencing/tcga (accessed on 10 February 2024)) in FPKM format, including samples from colon adenocarcinoma (COAD) and rectum adenocarcinoma (READ). Specimens without survival data were excluded, and the expression matrix was converted into TPM format. The gene expression profiles of six potential hub targets of HQD for anti-CRC liver metastasis were extracted from the TCGA dataset for least absolute shrinkage and selection operator (LASSO) Cox regression analysis using the ‘glmnet V4.1.8’ package. A risk score model was developed based on the appropriate variables, calculating risk scores for each patient according to Cox regression coefficients and the mRNA expression levels of selected genes. Patients were stratified into high- and low-risk score groups based on the median calculated risk score as the cutoff point. Kaplan–Meier (KM) survival analysis was performed to estimate overall survival differences between the two risk subgroups using the ‘SurvivalROC V1.0.3.1’ package.

##### Differentially Expressed Genes (DEGs) Screening Between the Two Subtypes and Functional Enrichment Analysis

To identify DEGs between the distinct risk score subgroups, the ‘limma V3.62.2’ package was utilized with criteria of *p* < 0.05 and |log2fold change (FC)| ≥ 0.2. A volcano plot was generated using the ‘tinyarray V2.4.3’ package to visualize DEGs. GO and KEGG functional enrichment analyses were conducted with the “clusterProfiler V4.14.6” package, defining *p* < 0.05 as statistically significant.

##### The Tumor Immune Microenvironment (TIME) Analysis

Five immune infiltration algorithms—TIMER, MCPcounter, EPIC, quantiseq, and CIBERSORT—were employed to assess CD8+ T cell levels in each tumor sample based on RNA-seq data from the TCGA database using the ‘IOBR V2.0.0’ package. Additionally, the functions of CD8+ T cells between the different populations were compared using the expression levels of cell-killing-related genes. The immune microenvironment, represented by MHC molecules, cytokine receptors, immunostimulatory factors, and chemokines, was examined between the risk score cohorts using data from the TISIDB database (http://cis.hku.hk/TISIDB/ (accessed on 22 April 2024)).

#### 4.4.5. Single-Cell RNA-Seq Data Analysis to Solidify Immunomodulatory Action of HQD

Public single-cell transcriptome data from GSE231559 were obtained from the GEO database, consisting of 8 normal liver tissues and 9 CRC liver metastasis tumor samples [61]. The ‘Seurat V5.2.1’ package was employed for dimensionality reduction, clustering, and visualization. The specific workflow is outlined in the Supplementary Materials and Methods.

Subsequently, single-cell metabolic activity analysis, intercellular communication analysis, trajectory analysis, and RNA velocity analysis were performed to elucidate the immunoregulatory mechanisms of HQD. The specific implementation details are provided in the Supplementary Materials and Methods.

#### 4.4.6. *In Vitro* Cellular Experimental Validation

##### Cell Viability Assay

A cell-counting kit 8 (CCK8; Shandong Sparkjade Biotechnology Co., Ltd., Jinan, China, CT0001) was employed to assess cell proliferation. The HCT116 and LoVo cell lines (human colon adenocarcinoma cell lines) were obtained from the Cell Resource Center, Peking Union Medical College (Beijing, China). HCT116 and LoVo cells were plated in 96-well plates at a density of 1500 cells per well and treated with naringenin at 300 μM for 0, 24, 48, or 72 h. Following treatment, the supernatant was removed, and 100 μL of DMEM or DMEM F12K containing 10 μL of CCK8 was added. The cells were then incubated at 37 °C for 4 h, and absorbance was measured at 450 nm.

##### Cell Migration and Invasion Experiments

For invasion assays, cells were incubated with naringenin at 300 μM for 48 h. An invasion chamber with 8 μm pores (Matrigel invasion chamber; Corning, Corning, NY, USA) was used to evaluate cell migration and invasion. In the invasion assay, 2 × 10^5^ cells in serum-free medium were added to the upper chamber, while 1 mL of 10% FBS DMEM or DMEM F12K was placed in the lower chamber. After 48 h, the number of cells that migrated was quantified by counting five random fields under a microscope (IX70; Olympus Corp., Tokyo, Japan). Similar methods were applied to assess migration without Matrigel coating, with five random fields counted for each chamber.

##### Glutamine Detection

For glutamine quantification, cells were plated in 6-well plates at a density of 1 × 10^5^ per well and incubated with naringenin at 300 μM for 48 h in 10% FBS DMEM or DMEM F12K. The supernatants were collected and centrifuged at 4000 rpm for 10 min at 4 °C, after which the supernatants were transferred into a microplate pre-coated with antibodies (50 μL per well). Standard glutamine solutions were added to create a standard dose-absorbance curve. Biotin-labeled glutamine was added to the wells for 1 h at 37 °C, followed by washing to remove supernatants. HRP-labeled streptavidin was then added (50 μL per well) for 30 min at 37 °C, with absorbance quantified at 450 nm using the substrate.

### 4.5. Statistical Analysis

Statistical analyses were performed using R statistical software V4.2.3or GraphPad Prism V8.0.1. Differences between two groups with normally distributed data were assessed using Student’s *t*-test; otherwise, the Mann–Whitney U test was employed. For multiple groups, a one-way analysis of variance (ANOVA) was conducted. All reported *p*-values were two-sided, with *p* < 0.05 considered statistically significant.

## 5. Conclusions

This study employs a comprehensive strategy to investigate the mechanisms of action of HQD against CRC, utilizing multimodal imaging, metabolomics, and network pharmacology. The findings were further validated through scRNA-seq, bioinformatics, molecular docking, molecular dynamics simulations, as well as AFM assays and cellular experiments. Results demonstrated that HQD effectively targets hub proteins such as GOT1, ABAT, and CYP1A2, inhibiting the exhaustion and regulatory phenotypes of cytotoxic T cells. This action reverses the immunosuppressive microenvironment and disrupts the glutamine metabolic pathway, thereby exerting its anti-CRC effects. In summary, this study not only provides novel insights into the theoretical foundation of HQD in CRC treatment but also establishes an efficient paradigm for further research into TCM as a potential alternative therapy for cancer prevention and treatment.

## Data Availability

The original contributions presented in the study are included in the article and Supplementary Materials; further inquiries can be directed to the corresponding author.

## Supplementary Materials

The following supporting information can be downloaded at: https://www.mdpi.com/article/10.3390/ph18071052/s1, Supplementary materials and methods and Figure S1: CellChat analysis to infer crosstalk among different cell types between normal liver and CRC liver metastasis; Table S1: Binding energy score between the key compounds and hub targets in HQD; Table S2: Binding free energy and energy decomposition terms calculated using the MMGBSA and MMPBSA methods. References [62,63,64,65,66,67,68,69] are cited in the supplementary materials.

## Abbreviations

The following abbreviations are used in this manuscript:

GOT1	Vesicle transport protein GOT1A
CYP1A2	Cytochrome P450 1A2
CA2	Carbonic anhydrase 2
HQD	Huang Qin Decoction
CRC	Colorectal cancer
scRNA-seq	Single-cell RNA sequence
TCM	Traditional Chinese Medicine
CAM	Complementary and alternative medicine
D4	Day 4
D12	Day 12
BLI	Bioluminescence imaging
MRI	Magnetic resonance imaging
QC	Quality control
TCMSP	Traditional Chinese Medicine Systems Pharmacology Database and Analysis Platform
OB	Oral bioavailability
DL	Drug-likeness
GEO	Gene Expression Omnibus
GO	Gene Ontology
KEGG	Kyoto Encyclopedia of Genes and Genomes
PPI	protein-protein interaction
PDB	Protein Data Bank
MD	Molecular dynamics
EM	Energy minimization
NVT	Isothermal and isovolume
NPT	Isothermal and isobaric
RMSD	Root mean square deviation
RMSF	Root mean square fluctuation
Rg	Radius of gyration
SASA	Solvent accessible surface area
MM/GBSA	Molecular Mechanics with Generalized Born and Surface Area
MM/PBSA	Molecular Mechanics Poisson-Boltzmann Surface Area
FEL	Gibbs free energy landscape
AFM	Atomic force microscope
TCGA	The Cancer Genome Atlas Program
COAD	Colon adenocarcinomas
READ	Rectal adenocarcinoma
TPM	Transcript per million
LASSO	Least absolute shrinkage and selection operator
KM	Kaplan–Meier
DEGs	Differential expressed genes
TIME	Tumor immune microenvironment
TIMER	Tumor Immune Estimation Resource
EPIC	Estimating the Proportion of Immune and Cancer Cells
CIBERSORT	Cell-type Identification by Estimating Relative Subsets of RNA Transcripts
UMI	Unique molecular identifier
OPLS-DA	Orthogonal partial least squares discriminant analysis
PCA	Principal component analysis
