# Supplementary material for: Integration of Untargeted Metabolomics, Network Pharmacology, Single-Cell RNA Sequencing, and Molecular Dynamics Simulation Reveals GOT1, CYP1A2, and CA2 as Potential Targets of Huang Qin Decoction Preventing Colorectal Cancer Liver Metastasis
Source: Pharmaceuticals (Basel). 2025 Jul 17;18(7):1052. doi: 10.3390/ph18071052 (PMC12299530; doi:10.3390/ph18071052)
Supplement: Supplementary file 1 [file pharmaceuticals-18-01052-s001.zip › pharmaceuticals-3740605-supplementary.pdf]

Supplementary materials and methods

Construction of a mouse model with CRC liver metastasis

Before surgery, shave the abdominal hair of the mouse with an electric clipper, followed disinfect using 75% ethanol. Then, the mouse was anesthetized and fixed on a metal plate with left lateral position. An incision was made under the left subcostal to expose the spleen, the spleens were pulled 1/2 to the outside of the incision. Draw up 100 μL SL4-luciferase murine colon cancer cells (1×10^6) diluted with PBS were slowly injected into the exposed spleen using a 26-gauge needle and then press the bleeding point with degreased cotton until no longer bleeds. Finally, suture the peritoneum and skin with stitch after place the spleen back.

Multimodal *in vivo* imaging to estimate mouse model of colon cancer with hepatic metastasis

To investigate the progression of tumor lesions in liver, tumor-bearing mouse underwent bioluminescence imaging and small animal MRI after inoculation. For bioluminescence imaging (BLI), mice were intraperitoneally injected with 150 mg/kg of D-Luciferin (Caliper Life Sciences，Alameda，CA，US) 3 minutes prior to imaging. Mice were anesthetized by isoflurane (2% vaporized in O_2) before and during imaging. *In vivo* BLI was performed using IVIS Spectrum CT (Xenogen, Perkin Elmer, MA, USA)，the detailed parameters as follows: field of view: 13.2, exposure time: auto, binning factor: 8. Bioluminescence intensity of regions of interest (ROIs) were measured as photon flux (p/sec/cm2/sr) using LivingImage software.

MRI scanning was performed on a 7.0 tesla small animal MRI equipment (PharmaScan 70/16 US, Bruker BioSpin, Ettlingen, Germany) with transmit/receive volume coil (Inner diameter: 40 mm). The mice were anesthetized by 2.5% isoflurane for induction and 1.5–2.0% for maintenance. MRI-compatible respiratory triggering system (PC-SAM, Small Animal Instruments Inc., Stony Brook, NY) was adopt during the whole experiment to remove the motion artifacts due to physiological movement.

Anatomical images of liver and spleen were acquired using a T2-TurboRARE sequence with the following parameters: repetition time 2500 ms, echo time 33 ms, field of view 40 × 40 mm, matrix size 256 × 256, flip angle 90°. Paravision 6.0.1 software was used for imaging data collection and processing.

Sample preparation for untargeted metabolomics

The tissue samples were homogenized three times in a mixed solvent of prechilled methanol/water/dichloromethane (1:1:0.7, V/V/V). The mixture was vortexed for 2 minutes and subsequently centrifuged at 15,000 rpm for 5 minutes at 4 °C. The lower layer was collected and dried using a vacuum lyophilizer. Finally, the dried powder was redissolved in 120 μL of acetonitrile (ACN)/deionized water (4:6, V/V), vortexed for 2 minutes, and centrifuged at 15,000 rpm for 10 minutes at 4 °C to obtain the supernatant for injection. Additionally, quality control (QC) samples were prepared by combining 10 μL of solvent from each test sample to monitor the repeatability and stability of the instrument[1].

Conditions for Chromatography and Mass Spectrometry

For chromatography, the mobile phase was consisted of aqueous 0.1% formic acid (A) and acetonitrile (B), the flow rate was 0.2 mL/min and the injection volume was 10.0 μL. The gradient elution conditions were set as follows: 0-3 min, 98-40% A, 3-10 min, 40% A, 10-12 min, 40-19% A, 12-20 min, 19% A, 20-22 min, 19-10% A, 22-32 min, 10% A, 32-33 min, 10-0% A, 33-35 min, 0% A. The total analytical time for each sample was 35 min.

The MS operation parameters were as follows, scan mode: FullMass+ddMS2, spray voltage: +3.5 /-3.2 kV, sheath gas flow rate: 40 PSI, aux gas flow rate: 11 Arb, capillary temperature: 350°C, aux gas heater temperature: 220°C, s- lens RF level: 55, scan range: 100-1000 m/z, resolution: 7000.

Data processing and analysis for metabolomics datasets

First, the raw data in .raw format were converted into mzXML format by Mass

Matrix MS Data File Conversion Tools (http://www.massmatrix.net/) and further processed with the on line open-source XCMS software (https://massconsortium.com/index-xcms-metlin.html) for peak recognition, alignment, area normalization, integration, as well as retention time correction to get metabolites feature matrix, which include retention time, *m/z* value, and peak area. Next, principal component analysis (PCA), orthogonal partial least squares-discriminant analysis (OPLS-DA) were conducted for multivariate statistical analysis to filter out the compounds with significant contribution to classification by R 4.2.3 software with 'ropls V1.40.0' package. Variables with variable importance in projection (VIP) > 1, $P < 0.05$ were regarded as differential metabolites. Identification of differential metabolites was conducted by comparing the MS and MS/MS mass spectrometry information with the online HMDB (https://hmdb.ca/), METLIN (https://metlin.scripps.edu/) databases. In addition, MetaboAnalyst 6.0 (https://www.metaboanalyst.ca/) was utilized to search for the KEGG enrichment pathways of differential metabolites, $P < 0.05$ was considered as significant enrichment pathways. Meanwhile, the gene-metabolite interaction network was also generated by import differential metabolites in network analysis module to explore and visualize of interactions between functionally related metabolites and genes.

GEO datasets processing

The GSE41258 dataset comprises 13 normal liver tissues and 47 CRC liver metastasis samples, based on the GPL96 platform (Affymetrix Human Genome U133A Array). The GSE38174 dataset includes 10 normal liver tissues and 30 CRC liver metastasis samples from the GPL6480 platform (Agilent-014850 Whole Human Genome Microarray 4x44K G4112F), while GSE14297 encompasses 5 normal liver tissues and 18 CRC liver metastasis samples subject to the GPL6370 platform (Illumina human-6 v2.0 expression beadchip). Differential gene expression analysis between normal and metastatic groups was performed using the 'limma V3.62.2' package in R 4.2.3. Genes with an adjusted $P < 0.05$ and $|\log2 \text{ fold change (FC)}| \geq 1$ were classified as differentially expressed genes (DEGs). Volcano plots were visualized using the

'ggplot2 V3.5.1' package in R 4.2.3.

Molecular dynamics (MD) simulation

The force fields for proteins and small molecule ligands were set to the CHARMM36 force field and CHARMM General Force Field (CGenFF), respectively[2]. A TIP3P water model was employed to simulate physiological conditions. The protocol included initial structure checks and topology file preparation for GROMACS, applying force parameters for proteins and ligands, followed by solvation to simulate a simple aqueous environment, which involved defining a water box and adding ions. Energy minimization (EM) was performed to ensure appropriate system geometry. Prior to dynamics simulation, 100 ps of NVT (isothermal and isovolume) and NPT (isothermal and isobaric) equilibrations were conducted to stabilize the temperature and pressure. An 80 ns molecular dynamics simulation was executed at room temperature (310 K) and 1 bar pressure, with trajectory data recorded every 2 ps.

Processing for Single cell dataset

Initially, the raw gene expression matrix in unique molecular identifier (UMI) count format was aggregated and converted into a Seurat object using the ‘Seurat V5.2.1’ R package. Genes detected in fewer than 3 cells, cells expressing fewer than 200 genes, and those with over 50% mitochondrial genes were excluded from subsequent analyses. The LogNormalize method was then applied to normalize the filtered data. Following this, 2,000 highly variable genes were identified for dimensionality reduction using the ‘FindVariableFeatures’ function with the variance stabilization transformation (vst) method. Scaling analysis and principal component analysis (PCA) were performed based on these highly variable genes. The ‘Harmony’ package was employed to merge the 17 Seurat objects and eliminate batch effects. The top 20 principal components (PCs) were selected to run ‘FindNeighbors’ and ‘RunUMAP’ for constructing a K-nearest neighbor graph. To identify distinct cell clusters, the ‘FindClusters’ function was executed with a resolution of 0.8. Cell subpopulations were visualized on a two-dimensional map using Uniform Manifold Approximation and Projection (UMAP) to

reveal cellular heterogeneity. The ‘FindAllMarkers’ function was used to identify feature gene panels for each cluster. Cell type assignments were based on canonical marker genes sourced from online databases: Cell Taxonomy (https://ngdc.cncb.ac.cn/celltaxonomy/)[3] and CellMarker 2.0 (http://117.50.127.228/CellMarker/index.html)[4].

Evaluation of single-cell metabolism activity

‘scMetabolism V0.2.1’ package[5] was employed to quantify the metabolic features at the single-cell resolution with VISION algorithm. Then, the variations in metabolic activity for each cell type between normal liver and hepatic metastases of CRC are visualized with radar map.

Intercellular communication analysis

Cell–cell communication was predicted with R package ‘CellChat V2.1.0’ based on previously known ligand-receptor interaction database CellChatDB[6]. Then, the total number of interactions and the strength of interactions between liver normal tissue and metastasis liver tumor of CRC were compared.

Trajectory and RNA velocity analysis

Monocle3 V1.3.7[7] was carried out to illustrate the cell state transition in subcluster of CD8+ T cell between liver normal and metastasis liver tumor of CRC. To summaries, the Seurat object of CD8+ T cell was extracted with ‘subset’ function, followed by cell re-clustering and cell type annotation. Next, the Seurat object was converted into CDS object for trajectory analysis using the ‘new_cell_data_set function’. After non-linear dimensionality reduction with UMAP and cluster cells, the pseudo-time was inferred and visualized with ‘plot_cells’ function.

RNA velocity methods leverage the information of unspliced and spliced transcripts to confirm the state of a single cell. Therefore, the first step is to extract the .loom file from the raw data with FASTQ format by velocyto command, which include pre-mature (unspliced) and mature (spliced) transcript information. Then, ‘scVelo V0.3.3’ package

was used to calculate RNA velocity values for each cell and visualize the result in UMAP embedding with python V3.11.5[8].

References

1. Wang, H.; Xu, J.; Chen, Y.; Zhang, R.; He, J.; Wang, Z.; Zang, Q.; Wei, J.; Song, X.; Abliz, Z. Optimization and Evaluation Strategy of Esophageal Tissue Preparation Protocols for Metabolomics by LC-MS. *Analytical chemistry* **2016**, *88*, 3459-3464, doi:10.1021/acs.analchem.5b04709.
2. Vanommeslaeghe, K.; Hatcher, E.; Acharya, C.; Kundu, S.; Zhong, S.; Shim, J.; Darian, E.; Guvench, O.; Lopes, P.; Vorobyov, I.; et al. CHARMM general force field: A force field for drug-like molecules compatible with the CHARMM all-atom additive biological force fields. *Journal of computational chemistry* **2010**, *31*, 671-690, doi:10.1002/jcc.21367.
3. Jiang, S.; Qian, Q.; Zhu, T.; Zong, W.; Shang, Y.; Jin, T.; Zhang, Y.; Chen, M.; Wu, Z.; Chu, Y.; et al. Cell Taxonomy: a curated repository of cell types with multifaceted characterization. *Nucleic acids research* **2023**, *51*, D853-d860, doi:10.1093/nar/gkac816.
4. Hu, C.; Li, T.; Xu, Y.; Zhang, X.; Li, F.; Bai, J.; Chen, J.; Jiang, W.; Yang, K.; Ou, Q.; et al. CellMarker 2.0: an updated database of manually curated cell markers in human/mouse and web tools based on scRNA-seq data. *Nucleic acids research* **2023**, *51*, D870-d876, doi:10.1093/nar/gkac947.
5. Wu, Y.; Yang, S.; Ma, J.; Chen, Z.; Song, G.; Rao, D.; Cheng, Y.; Huang, S.; Liu, Y.; Jiang, S.; et al. Spatiotemporal Immune Landscape of Colorectal Cancer Liver Metastasis at Single-Cell Level. *Cancer discovery* **2022**, *12*, 134-153, doi:10.1158/2159-8290.Cd-21-0316.
6. Jin, S.; Guerrero-Juarez, C.F.; Zhang, L.; Chang, I.; Ramos, R.; Kuan, C.-H.; Myung, P.; Plikus, M.V.; Nie, Q. Inference and analysis of cell-cell communication using CellChat. *Nature Communications* **2021**, *12*, 1088, doi:10.1038/s41467-021-21246-9.
7. Packer, J.S.; Zhu, Q.; Huynh, C.; Sivaramakrishnan, P.; Preston, E.; Dueck, H.; Stefanik, D.; Tan, K.; Trapnell, C.; Kim, J.; et al. A lineage-resolved molecular atlas of C. elegans embryogenesis at single-cell resolution. *Science (New York, N.Y.)* **2019**, *365*, doi:10.1126/science.aax1971.
8. La Manno, G.; Soldatov, R.; Zeisel, A.; Braun, E.; Hochgerner, H.; Petukhov, V.; Lidschreiber, K.; Kastriti, M.E.; Lönnerberg, P.; Furlan, A.; et al. RNA velocity of single cells. *Nature* **2018**, *560*, 494-498, doi:10.1038/s41586-018-0414-6.

Supplementary figures

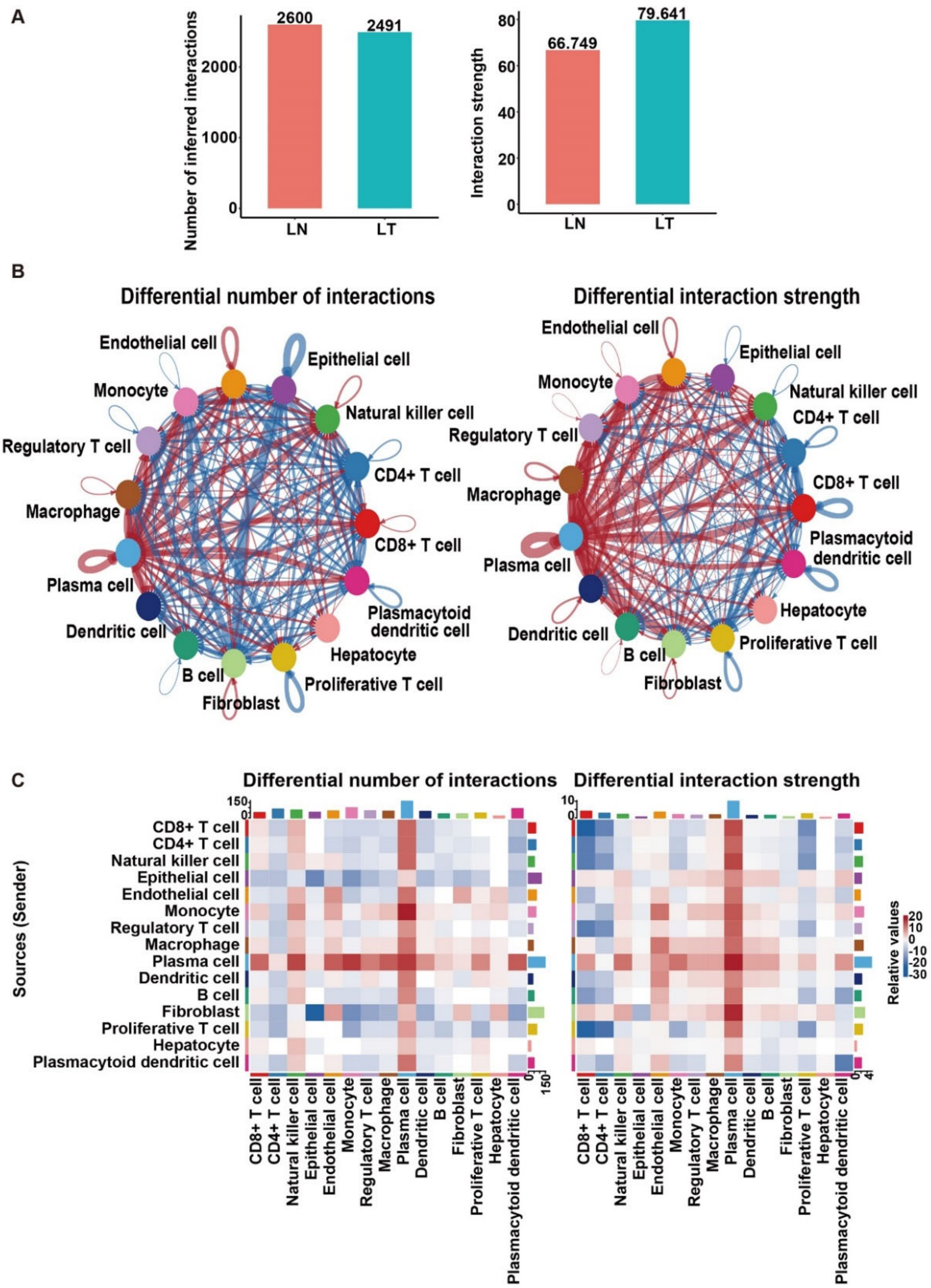

Figure S1 CellChat analysis to infer crosstalk among different cell types between normal liver and CRC liver metastasis. (**A**) A bar plot illustrating the inferred number and strength of intercellular communications. (**B**) Circle plots showing the differential number (left) and weight (right) of interactions within the cell-cell link network among the identified cell lineages. (**C**) Heatmaps depicting the efferent and afferent contributions, leading to distinct interaction numbers (left) and interaction strengths (right) between CRC liver metastasis and adjacent normal liver tissue.

Table S1. Binding energy score between the key compounds and hub targets in HQD.

Targets	Compound ID	Chemical name	No. CAS	Affinity (Kcal/mol)
ABAT	HQD049	naringenin	480-41-1	−7.7
CYP1A2	HQD123	quercetin	117-39-5	−9.7
	HQD136	beta-carotene	7235-40-7	−8.5
	HQD013	oroxylin a	480-11-5	−9.9
	HQD037	kaempferol	520-18-3	−10
CYP3A4	HQD013	oroxylin a	480-11-5	−8.9
	HQD037	kaempferol	520-18-3	−8.8
	HQD123	quercetin	117-39-5	−9.1
	HQD126	coumestrol	479-13-0	−9
	HQD136	beta-carotene	7235-40-7	−9.3
GOT1	HQD049	naringenin	480-41-1	−7.6
CA2	HQD030	5,7,4'-trihydroxy-6-methoxyflavanone	94942-49-1	−7.39
	HQD031	5,7,4'-trihydroxy-8-methoxyflavanone	57096-02-3	−7.05
	HQD032	rivularin	70028-59-0	−6.86

	HQD056	(E)-1-(2,4-dihydroxyphenyl)-3-(2,2-dimethylchromen-6-yl) prop-2-en-1-one	155233-19-5	−8.43
	HQD063	Glypallichalcone	146763-58-8	−7.44
	HQD065	Licochalcone B	58749-23-8	−7.09
	HQD101	licochalcone a	58749-22-7	−8.26
	HQD131	(S)-Coclaurine	486-39-5	−7.77
	HQD133	Stepholidine	16562-13-3	−6.5
CYP2C9	HQD013	oroxylin a	480-11-5	−8.6

Table S2 Binding free energy and energy decomposition terms calculated using the MMGBSA and MMPBSA methods.

Energy Component	CA2	CYP1A2	GOT1
	**Average binding energy ± Standard Deviation (Kcal/mol)**	**Average binding energy ± Standard Deviation (Kcal/mol)**	**Average binding energy ± Standard Deviation (Kcal/mol)**
**MMGBSA**			
VDWAALS	−10.48 ± 0.1	−36.61 ± 0.03	−21.44 ± 1.15
EEL	−3.27 ± 3.52	−17 ± 0.2	−24.93 ± 1.44
EGB	9.15 ± 0.02	32.82 ± 0.81	34.33 ± 0.1
ESURF	−1.57 ± 0.74	−4.79 ± 0.01	−3.38 ± 0.02
GGAS	−13.75 ± 3.58	−53.61 ± 0.2	−46.37 ± 1.85
GSOLV	7.58 ± 0.74	28.04 ± 0.81	30.96 ± 0.1
TOTAL	−6.17 ± 3.66	−25.57 ± 0.84	−15.41 ± 1.85
**MMPBSA**			
VDWAALS	−10.48 ± 0.1	−36.35 ± 0.58	−19.5 ± 0.62
EEL	−3.27 ± 3.52	−14.49 ± 2.11	−12.9 ± 1.84
EPB	8.18 ± 0.36	37.59 ± 0.79	24.18 ± 0.08
ENPOLAR	−1.45 ± 0.74	−3.62 ± 0.02	−2.6 ± 0.03
GGAS	−13.75 ± 3.58	−50.84 ± 2.22	−32.4 ± 2.01
GSOLV	6.73 ± 0.82	33.97 ± 0.79	21.58 ± 0.09
TOTAL	−7.02 ± 3.67	−16.87 ± 2.36	−10.81 ± 2.01

VDWAALS: van der Waals energy, EEL: electrostatic energy, EGB/EPB: polar solvation energy calculated by PB or GB, respectively, ESURF/ ENPOLAR: non-polar solvation energy calculated by PB or GB, GGAS: gas-phase free energy, GSOLV: solvation energy.
